# Oxidation of Atg3 and Atg7 mediates inhibition of autophagy

**DOI:** 10.1038/s41467-017-02352-z

**Published:** 2018-01-08

**Authors:** Karen Frudd, Thomas Burgoyne, Joseph Robert Burgoyne

**Affiliations:** 1 King’s College London, Cardiovascular Division, The British Heart Foundation, Centre of Excellence, The Rayne Institute, St Thomas’ Hospital, London, SE1 7EH UK; 2 Electron Microscopy Unit, Royal Brompton Hospital, Sydney Street, London, SW3 6NP UK; 3 National Heart and Lung Institute, Imperial College, Dovehouse Street, London, SW3 6LR UK

## Abstract

Macroautophagy (autophagy) is a crucial cellular stress response for degrading defective macromolecules and organelles, as well as providing bioenergetic intermediates during hypoxia and nutrient deprivation. Here we report a thiol-dependent process that may account for impaired autophagy during aging. This is through direct oxidation of key autophagy-related (Atg) proteins Atg3 and Atg7. When inactive Atg3 and Atg7 are protected from oxidation due to stable covalent interaction with their substrate LC3. This interaction becomes transient upon activation of Atg3 and Atg7 due to transfer of LC3 to phosphatidylethanolamine (lipidation), a process crucial for functional autophagy. However, loss in covalent-bound LC3 also sensitizes the catalytic thiols of Atg3 and Atg7 to inhibitory oxidation that prevents LC3 lipidation, observed in vitro and in mouse aorta. Here findings provide a thiol-dependent process for negatively regulating autophagy that may contribute to the process of aging, as well as therapeutic targets to regulate autophagosome maturation.

## Abstract (web summary)

A dysfunction of autophagy can be detected in aged tissues, but how this is regulated is unclear. Here, the authors show in vitro and in aged mice aorta, that inhibition of LC3 lipidation under conditions of oxidative stress causes oxidation of Atg3 and Atg7, preventing autophagosome maturation.

## Introduction

Macroautophagy (hereafter referred to as autophagy) is a catabolic process that delivers cellular components to lysosomes for degradation. This process of removing aged or dysfunctional macromolecules and organelles is critical in maintaining cellular homeostasis. The regulation of the autophagy pathway is controlled by the gatekeeper protein, mammalian target of rapamycinm (mTOR)^1^. Inhibition of mTOR by energy deprivation, hypoxia, or amino acid withdrawal allows autophagosome nucleation, by activating unc-51 like autophagy activating kinase 1 (ULK1), and releasing beclin1 from its inhibitory complex with B-cell lymphoma 2 (Bcl-2)^2,3^. Once nucleated the maturation of the growing autophagosome requires the activities of two conjugation pathways. Firstly, the E1-like and E2-like enzymes Atg7 and Atg10 constitutively conjugate Atg5 to Atg12 through a glycyl lysine isopeptide bond^4^. The covalently bound Atg5 and Atg12 then acts as an E3-like enzyme in the second conjugation pathway, where LC3 is attached to phosphatidylethanolamine (PE). The conjugation of LC3 to PE (LC3II) is mediated by the E1-like and E2-like enzymes Atg7 and Atg3 respectively^5^. This process occurs once autophagy is initiated, as Atg3 is regulated by a membrane-curvature-sensing domain, which switches on enzyme activity only at highly curved membrane surfaces, characteristic of a growing autophagosome^6^. As conjugation of LC3 to PE only occurs after induction of autophagy, its detection has become the gold standard for measuring activation of this catabolic process. However, the conjugation of PE to LC3 is also involved in autophagosome maturation as it contributes to cargo recognition, membrane extension, and closure of the autophagosome membrane^7,8^. More importantly, LC3 is also crucial in autophagosome−lysosome fusion and for degradation of the inner autophagosomal membrane^9,10^. The importance of LC3 lipidation is underscored by the neonatal lethality of knocking out either Atg3 or Atg7^11^. In addition, conditional knockout of Atg3 or Atg7 impairs autophagosome formation and starvation-induced degradation of proteins and organelles^12,13^. The loss in autophagic renewal is also a key feature of aging that leads to accumulation of damaged proteins and organelles, resulting in cellular dysfunction. This explains why model organisms with decreased autophagy have accelerated aging, whereas enhanced stimulation may have potent anti-aging and disease-preventing effects^14^. Here we describe a molecular mechanism for the acute inhibition of amino acid starvation-induced LC3 lipidation, under conditions of oxidative stress due to the direct oxidation of Atg3 and Atg7. This is highly relevant as perturbations in cellular redox have been causatively linked to aging and the pathogenesis of many diseases, which are also broadly characterized by dysregulated autophagy^15–21^. In addition, stable covalent complexes between LC3 and both Atg3 and Atg7 were identified and found to decrease upon autophagy induction, which sensitized these enzymes to oxidative inhibition. This thiol-dependent process for inhibiting LC3 lipidation was also observed in aged mouse aorta, thus likely causative in decreasing the ability to stimulate autophagy during starvation. Therefore, our findings provide insights into the regulation of autophagy, but also a process that is likely to contribute to the process of aging.

## Results

### Rapid oxidative inhibition of LC3 lipidation

**Fig. 1:**
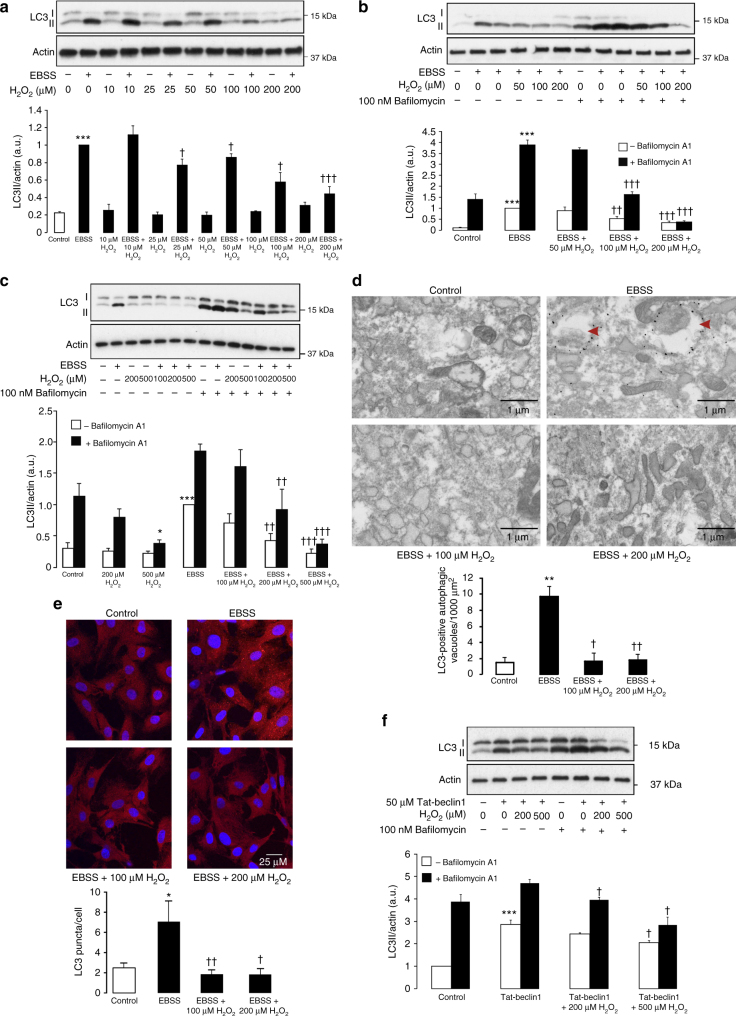
LC3 lipidation is inhibited by H_2_O_2_. **a** The withdrawal of amino acids from SMC for 1 h induces LC3 lipidation that decreased in a concentration-dependent manner by H_2_O_2_ co-treatment. The graph below shows quantification of LC3 lipidation from treated SMC. **b** Loss of LC3 lipidation in amino-acid-starved SMC is maintained when fusion between autophagosomes and lysosomes is inhibited by bafilomycin A1. The graph below shows quantification of LC3 lipidation from treated SMC in the presence or absence of bafilomycin A1. **c** In HEK cells H_2_O_2_ also attenuates LC3 lipidation in response to 1 h amino acid withdrawal in the presence or absence of bafilomycin A1. The graph below shows quantification of LC3 lipidation from treated HEK cells. **d** Electron microscopy of immunogold-labeled LC3 shows loss of LC3II-positive autophagic vacuoles (arrows) in amino-acid-starved SMC exposed to H_2_O_2_. The graph below shows quantification of LC3II-positive autophagic vacuoles in treated SMC. **e** Fluorescence microscopy of LC3 shows increased puncta formation in amino-acid-starved SMC that is lost with H_2_O_2_ co-treatment. The graph below shows quantification of LC3 puncta from treated SMC. **f** Direct induction of autophagosome nucleation in HEK cells by tat-beclin1 peptide induces LC3 lipidation, which is inhibited by H_2_O_2_ treatment in the presence or absence of bafilomycin A1. The graph below shows quantification of LC3 lipidation from HEK cells treated with tat-beclin1. All data represent mean ± s.e. from 3 (**a**, **b**, **f**) or 4−7 (**c**) independent experiments. *P < 0.05, **P < 0.01, ***P < 0.005 statistical significance relative to control or bafilomycin A1 alone. †*P* < 0.05, ††*P* < 0.01, †††*P* < 0.005 statistical significance (Dunnett’s test) relative to EBSS, tat-beclin1 or either combined with bafilomycin A1

The formation of LC3II induced by amino acid starvation in human embryonic kidney 293 (HEK) cells and rat aortic smooth muscle cells (SMC) was dose-dependently inhibited by H_2_O_2_ treatment (Figs. 1a–c). This loss in LC3 lipidation due to increased intracellular reactive oxygen species (ROS) did not impact on cell viability, which was enhanced by the rapid inducer of apoptosis raptinal (Supplementary Fig. 1A–F)^22^. Therefore, impaired LC3 lipidation by H_2_O_2_ was likely mediated by direct inhibition of the autophagy pathway. Consistent with these findings we observed an increase in the localization of LC3 to autophagic vacuoles in SMC during amino acid starvation, which then was attenuated by H_2_O_2_ co-treatment (Figs. 1d, e, Supplementary Fig. 2A, B). In addition, H_2_O_2_ co-treatment also decreased autophagic vacuole formation (Supplementary Fig. 2C). To further assess the loss in LC3 lipidation with H_2_O_2_ treatment we used the late phase inhibitor of autophagy bafilomycin A1. Bafilomycin A1 increased the accumulation of starvation-induced LC3II that remained inhibited by H_2_O_2_, meaning a loss in PE conjugation was responsible, as opposed to increased turnover of LC3II. The inhibition of LC3 lipidation by H_2_O_2_ following amino acid withdrawal persisted over several hours and, consistent with loss in autophagy, prevented starvation-induced lysosomal degradation of p62 (Supplementary Fig. 3A). In addition, as an acute 30-min treatment with H_2_O_2_ was sufficient to block autophagy (Supplementary Fig. 3B), this suggested the effect was due to direct enzyme oxidation rather than transcriptional regulation. Certainly, this was consistent with LC3A gene expression, which remained unaltered by H_2_O_2_ treatment (Supplementary Fig. 3C). In addition, this process was independent of protein translation or proteasomal degradation, as amino-acid-starved cells co-treated with cycloheximide or MG132 had impaired LC3II formation when exposed to H_2_O_2_ (Supplementary Fig. 3D, E).

### Atg3 and Atg7 form stable covalent complexes with LC3

**Fig. 2:**
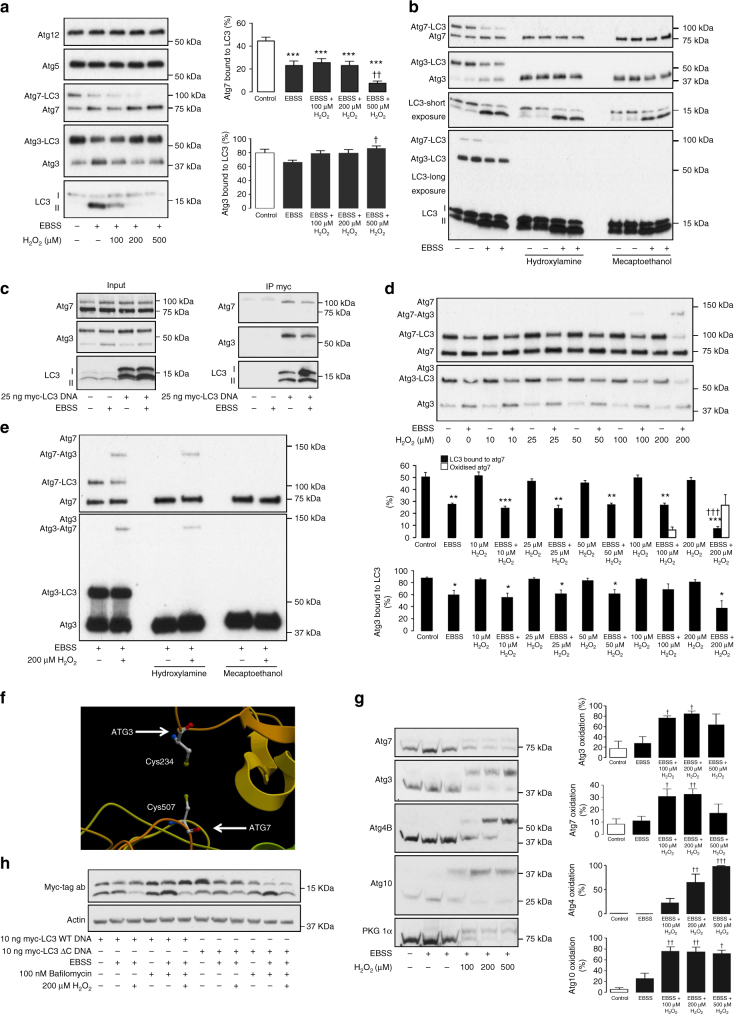
Atg3 and Atg7 form stable covalent intracellular complexes with LC3 and are sensitive to catalytic thiol oxidation. **a** In HEK cells the conjugation of Atg5 and Atg12 remained unaffected by amino acid withdrawal or co-treatment with H_2_O_2_. However, Atg3 and Atg7 are able to form stable covalent complexes with LC3 that decrease during amino acid withdrawal. The graphs to the right show quantification of Atg3-LC3 and Atg7-LC3 from treated HEK cells. **b** The higher molecular weight complexes for Atg3 and Atg7 can also be observed in HEK cells when immunoblotted for LC3, and are selectively hydrolyzed by hydroxylamine or reduced by beta-mercaptoethanol. **c** LC3 covalently bound to Atg3 and Atg7 is co-purified when myc-tagged LC3 is immunoprecipitated. **d** Stable covalent complexes between LC3 and either Atg3 or Atg7 can also be observed in SMC, where it decreases upon amino acid withdrawal. In addition, when amino acids are withdrawn Atg7 is able to form a higher molecular weight (~130 kDa) oxidized complex in the presence of H_2_O_2_. The graphs below show quantification of Atg3-LC3 and Atg-LC3 from treated SMC. **e** The higher molecular weight oxidized complex detected for Atg7 at approximately 130 kDa can also be observed for Atg3 on a higher exposure and is reducible only with beta-mercaptoethanol, thus supporting the potential formation of an intermolecular disulfide bond between these two enzymes when exposed to H_2_O_2_. **f** Structure of the yeast complex between Atg3 and Atg7 (4GSL) showing close proximity of catalytic thiols. **g** In HEK cells oxidation of Atg3 and Atg7 can be observed in amino-acid-starved cells exposed to H_2_O_2_, detected by a mobility shift following the PEG-switch assay. The graphs to the right show quantification of Atg3, Atg7, Atg4, Atg10, and PKGIα oxidation from treated HEK cells. **h** Amino acid deprivation induced lipidation of overexpressed wild-type and C-terminal truncated LC3 (ΔC), which was attenuated by H_2_O_2_ in the presence or absence of bafilomycin. All data represent mean ± s.e. from 5–8 (**a**) or 3 (**d**, **g**) independent experiments. *P < 0.05, **P < 0.01, ***P < 0.005 statistical significance (Dunnett’s test) relative to control. †*P* < 0.05, ††*P* < 0.01, †††*P* < 0.005 statistical significance relative to EBSS treatment

Activation of autophagy with the cell-permeable peptide tat-beclin1, bypassing mTOR regulation, was also blocked by H_2_O_2_ without compromising cell viability, thus placing the inhibitory mechanism that is rationally mediated by protein oxidation downstream of beclin1 (Fig. 1f, Supplementary Fig. 3F–H)^23^. Consequently, we next assessed the conjugation systems that lay downstream of beclin1 responsible for the lipidation of LC3^24^, searching for the target protein(s) that are oxidized by H_2_O_2_, as they would likely mediate the inhibition of autophagy. A key way in which proteins can be regulated by oxidants is through induction of regulatory disulfide bonds, including interprotein complex formation^25^. Formation of these complexes in response to H_2_O_2_ was assessed using non-reducing sodium dodecyl sulfate polyacrylamide gel electrophoresis (SDS-PAGE), which keeps any such disulfides that may mediate the inhibition of LC3II formation intact. Here we found the constitutive thiol-dependent conjugation of Atg5 to Atg12 needed for LC3 lipidation was unaffected by H_2_O_2_ treatment in HEK cells (Fig. 2a)^26^. This is perhaps unsurprising considering how rapidly H_2_O_2_ inhibited LC3II formation. However, on assessing Atg3 and Atg7 in HEK cells under non-reducing conditions, we observed higher molecular weight complexes (approximately an additional 15 kDa) consistent with covalent-bound LC3 (Fig. 2a). This was corroborated by a previous study showing the yeast homolog of LC3, Atg8, can form stable intracellular thioester complexes with Atg3 and Atg7^5^. The identity of higher molecular complexes for Atg3 and Atg7 were substantiated as they were also observed on an LC3 immunoblot (Fig. 2b). These complexes were due to a thio-ester linkage as they could be hydrolyzed by hydroxylamine. In addition, the thio-ester bond between LC3 and either Atg3 or Atg7 could be cleaved by the thiol-reducing agent beta-mercaptoethanol. The identity of the higher molecular weight complexes was further supported by co-immunoprecipitation studies. Here higher molecular weight forms of Atg3 and Atg7 were co-purified when myc-tagged LC3 was immunoprecipitated, thus further substantiating stable covalent interaction with LC3 (Fig. 2c). The higher molecular weight covalent complexes observed for Atg3 and Atg7 comprised of LC3A and LC3B, as well as the essential autophagy regulators GABA(A) receptor-associated protein-like (GABARAPL) isoforms 1 and 2 (Supplementary Fig. 4A). Both GABARAPL1 and 2 formed complexes at molecular weights consistent with bound Atg3 and Atg7, which decreased following an hour of amino acid withdrawal and could be selectively cleaved using hydroxylamine. Consistent with the oxidative inhibition of enzymes involved in LC3II formation, the lipidation of GABARAPL1 by amino acid deprivation was also attenuated by H_2_O_2_ (Supplementary Fig. 4B).

### Atg3 and Atg7 are susceptible to catalytic thiol oxidation

The quantity of covalent-bound LC3 decreased in HEK cells and SMC during amino acid withdrawal due to activation of Atg3 and Atg7 (Figs. 2a, d). In addition, after H_2_O_2_ treatment loss in stable covalent LC3 complexes following amino acid withdrawal was sustained in SMC. This is consistent with maintained upstream stimulation of the Atg3 and Atg7 complex under oxidizing conditions, but potentially decreased enzymatic activity due to direct oxidation. Therefore, as these enzymes are end-mediators in conjugating PE to LC3, this indicates that they are the likely targets of oxidation responsible for H_2_O_2_-dependent inhibition of LC3 lipidation. This was supported by the H_2_O_2_-dependent formation of an intermolecular disulfide complex between Atg3 and Atg7 in SMC only after amino acid starvation (Figs. 2d, e). The select formation of a disulfide complex after amino acid starvation is consistent with increased thiol availability on Atg3 and Atg7, due to a decrease in covalent LC3 interaction. The oxidized complex observed for Atg3 and Atg7 was at the same molecular weight, and with a mass consistent for a covalent heterodimer comprising of each protein (Fig. 2e). The identity of the conjoining bond was confirmed as intermolecular disulfide, as it was lost with beta-mercaptoethanol but not hydroxylamine treatment. In addition, consistent with oxidation occurring at the active site cysteine of Atg3 and Atg7, the oxidized complex did not contain LC3, as it resolved at the same molecular weight after hydroxylamine treatment. The formation of an intermolecular disulfide between Atg3 and Atg7 is consistent with the crystal structure of the yeast complex, where the proximal exposed catalytic cysteine of each enzyme is closely aligned, allowing LC3 transfer, but also disulfide formation (Fig. 2f)^27^. Although disulfide dimerization of Atg3 and Atg7 was not observed in HEK cells, likely due to increased LC3 occupancy on Atg3, reversible oxidation still occurred and was detected using the PEG-switch assay (Fig. 2g). The extent of reversible Atg3 and Atg7 oxidation in H_2_O_2_-treated HEK cells was comparable to that of the known redox sensor, cyclic guanosine monophosphate (cGMP)-dependent protein kinase 1 alpha (PKG1α)^28^. In addition to Atg3 and Atg7, Atg4B and Atg10 were also found to be sensitive to oxidation. However, oxidation of Atg10 is unlikely to impair LC3 lipidation under the experimental conditions studied, as H_2_O_2_ treatment did not impact on the conjugation of Atg5 to Atg12 (Fig. 2a, Supplementary Fig. 5A). Also, the oxidation of Atg4B previously described^29^ is unlikely to explain loss in LC3 lipidation following acute H_2_O_2_ treatment, due to constitutive enzyme activity^30^. Here loss in LC3 lipidation was independent of Atg4B oxidation, as a truncated LC3 (ΔC) mutant not requiring protease activity, underwent amino-acid-starved lipidation, which was attenuated by H_2_O_2_ to the same extent as wild-type protein (Fig. 2h). Therefore, loss in LC3 lipidation is likely due to oxidation of Atg3 or Atg7. This is consistent with the select activation of Atg3 and Atg7 upon stimulation of autophagy, with disulfide formation or potential S-glutathiolation preventing interaction and lipidation of LC3.

### Oxidation of Atg3 and Atg7 prevents LC3 lipidation

**Fig. 3:**
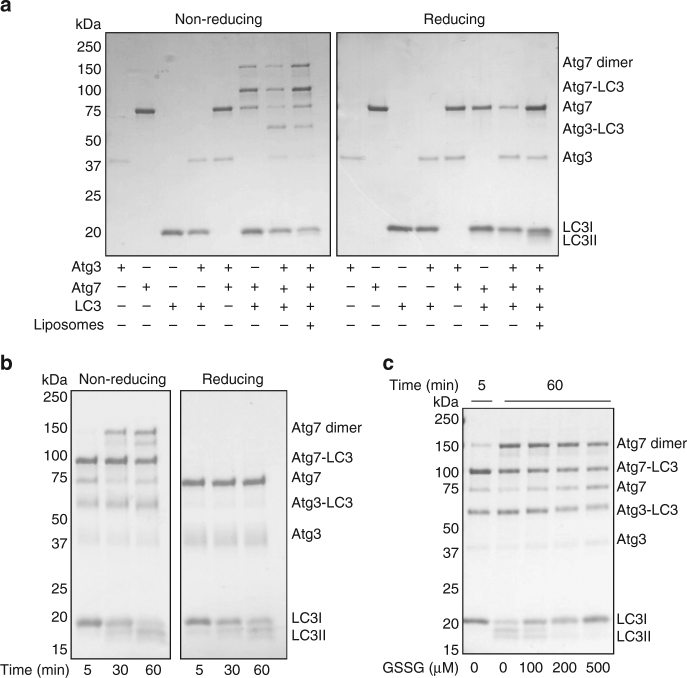
Atg3 and Atg7 form stable covalent complexes with LC3 and are sensitive to oxidative inhibition. **a** Reducible higher molecular weight complexes corresponding to LC3-bound Atg3 and Atg7 can be observed for recombinant enzymes, in addition to a disulfide-bound Atg7 dimer. The formation of a stable complex between LC3 and Atg3 requires Atg7, and liposomes are needed for LC3 lipidation. **b** The lipidation of LC3 in a complete reaction mixture increases over 60 min and stable complexes with Atg3 or Atg7 can be reduced by beta-mercaptoethanol. **c** Oxidized glutathione (GSSG) inhibited the lipidation of LC3 in a complete reaction mixture, and decreased LC3 covalent-bound to Atg3 and Atg7

To further assess in greater detail the impact of protein oxidation on LC3II formation, we performed in vitro lipidation assays^6^. Once again, we observed higher molecular weight complexes for Atg3 and Atg7 consistent with their stable covalent interaction with LC3 under non-reducing conditions (Fig. 3a), which were lost with beta-mercaptoethanol treatment. In addition, we observed a higher molecular weight complex when only Atg7 and LC3 were present, which had a mass consistent with an oxidized Atg7 disulfide dimer. The formation of an Atg7 disulfide dimer is likely an artifact of its high concentration in an in vitro model system, as the oxidized complex observed in SMC was of a lower molecular weight and comprised of both Atg3 and Atg7. However, the formation of a stable complex between Atg3 and LC3 was dependent on Atg7. This is consistent with the proposed mechanism of LC3 lipidation, where LC3 binds to Atg7 before being transferred to Atg3, where it is then conjugated to PE^5^. In a complete cell-free reaction mixture, the lipidation of LC3 increased over 60 min (Fig. 3b, Supplementary Fig. 5B). As well as lipidated LC3, an additional form (middle band) can also be observed that is likely an adenylated intermediate^6^. Here the increase in LC3 lipidation in a complete cell-free reaction was inhibited by S-glutathiolation, induced by the addition of oxidized glutathione (GSSG) (Fig. 3c, Supplementary Fig. 5C). The addition of GSSG also led to increased accumulation of unbound Atg3 and Atg7, consistent with catalytic cysteine oxidation through enhanced S-glutathiolation.

### Identifying sites of Atg3 and Atg7 oxidation

**Fig. 4:**
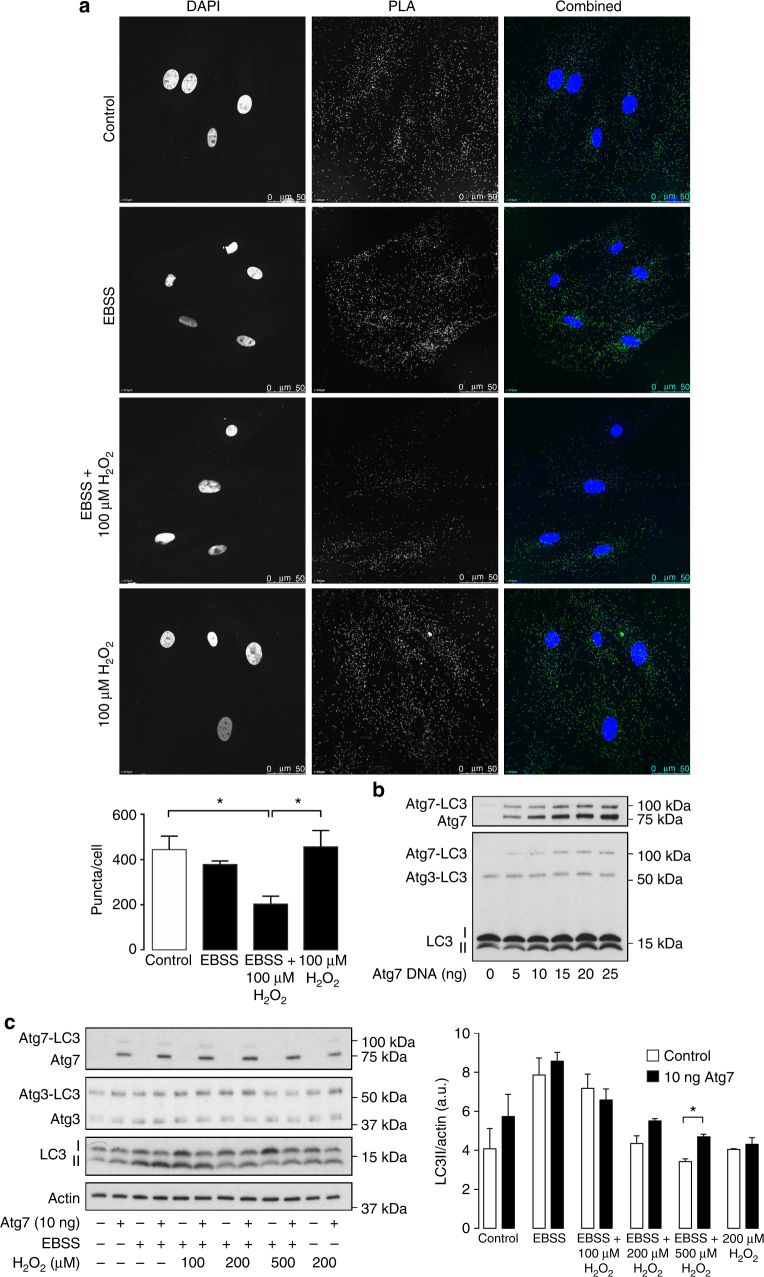
Atg7 overexpression attenuates oxidant-dependent loss in LC3 lipidation. **a** The interaction between Atg3 and Atg7 detected using a PLA assay did not change upon amino acid withdrawal. However, H_2_O_2_ treatment prevented interaction only in amino-acid-deprived cells, thus consistent with direct oxidation preventing Atg3 and Atg7 interaction. **b** Atg7 overexpression increases the quantity of LC3 that is covalently bound to Atg7, detected on Atg7 and LC3 immunoblots. **c** Atg7 overexpression preserves the lipidation of LC3 in amino-acid-starved cells treated with H_2_O_2_. The graph to the right shows quantification of LC3 lipidation from treated mock or Atg7 transfected HEK cells. All data represent mean ± s.e. from three independent experiments. *P < 0.05 statistical significance (Dunnett’s test)

**Fig. 5:**
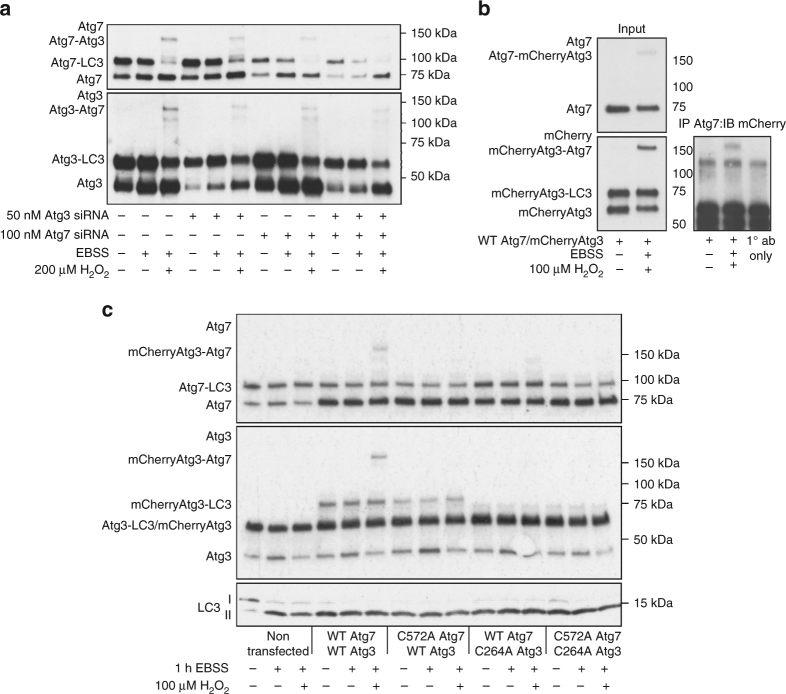
Under pro-oxidizing conditions a disulfide forms between the catalytic thiols of Atg3 and Atg7. **a** siRNA knock-down of Atg7 attenuates intermolecular disulfide oxidation of Atg3, and loss of Atg3 prevents Atg7 disulfide dimerization. **b** Immunoprecipitation of Atg7 leads co-purification of disulfide-bound mCherry-Atg3. **c** Overexpressed wild-type mCherryAtg3 and wild-type Atg7 form a disulfide-bound heterodimer in amino-acid-starved cells treated with H_2_O_2_. However, mutation of the catalytic thiol to an alanine on either Atg3 or Atg7 prevents intermolecular disulfide formation

The interaction of Atg3 and Atg7 is required for LC3 lipidation as it positions the catalytic thiol of each enzyme into close proximity to allow transfer of LC3^27^. Therefore, we next assessed if interaction between Atg3 and Atg7 was impacted by enzyme oxidation. In fed cells and those deprived of amino acids, a large proportion of interacting Atg3 and Atg7 could be observed using a proximity ligation assay (Fig. 4a). In amino-acid-deprived cells exposed to H_2_O_2_, there was a loss in Atg3 and Atg7 interaction. However, this interaction was unaffected by H_2_O_2_ in cells cultured in complete media. These findings are consistent with a loss in covalent LC3 interaction that exposes catalytic thiols on Atg3 and Atg7 to oxidation, with oxidative modification preventing physical enzyme interaction. As oxidation of Atg3 and Atg7 prevents LC3 lipidation, we next attempted to prevent this process by increasing the pool of reduced Atg7. The overexpression of Atg7 in HEK cells led to an increase in covalent-bound LC3 (Fig. 4b). This further substantiated the presence of a stable covalent complex between these proteins, as its formation directly correlated between Atg7 and LC3 immunoblots. In addition, this supported the functional activity of overexpressed Atg7. As anticipated, HEK cell overexpressing Atg7 were significantly protected from a loss in LC3 lipidation in amino-acid-deprived cells exposed to H_2_O_2_ (Fig. 4c). Furthermore, the formation of a disulfide-bound heterodimer between Atg3 and Atg7, under oxidizing conditions, was substantiated by siRNA knock-down of either enzyme. The knock-down of Atg3 attenuated Atg7 disulfide formation, and loss of Atg7 prevented the disulfide dimerization of Atg3 (Fig. 5a). Furthermore, immunoprecipitation of Atg7 led to co-purification of the higher molecular weight oxidized complex for mCherry-Atg3, thus confirming formation of a disulfide-bound heterodimer (Fig. 5b). To confirm if catalytic thiols on Atg3 and Atg7 were sites of oxidation responsible for formation of the disulfide-bound heterodimer, we generated cysteine to alanine mutants. In transfected SMC, an intermolecular disulfide could be observed between wild-type mCherry-Atg3 and wild-type Atg7 after amino acid starvation and H_2_O_2_ treatment (Fig. 5c, Supplementary Fig. 5D). However, mutation of either the catalytic thiol on Atg3 (C264A) or Atg7 (C572A) prevented disulfide formation, thus confirming the active site thiols as the targets of oxidation.

### Endogenous cellular oxidants inhibit LC3 lipidation

**Fig. 6:**
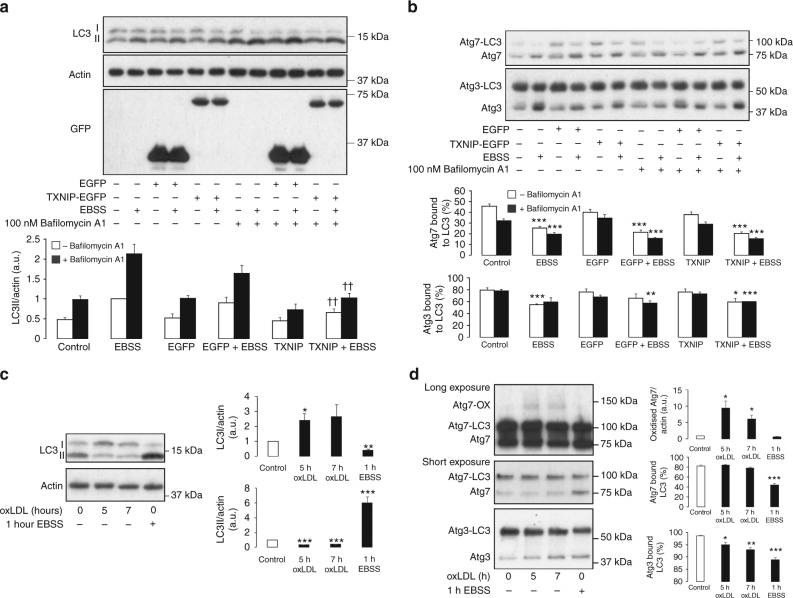
Inhibition of the thioredoxin-reducing system or oxLDL treatment prevents LC3 lipidation. **a** The overexpression of TXNIP in HEK cells but not EGFP significantly attenuates LC3 lipidation in response to amino acid withdrawal, observed in the presence or absence of bafilomycin A1. The graph below shows quantification of LC3 lipidation from treated EGFP or TXNIP transfected HEK cells. **b** During amino acid withdrawal the loss in LC3 interaction with Atg3 and Atg7 is maintained in cells overexpressing TXNIP or EGFP. The graphs below show quantification of Atg3-LC3 and Atg7-LC3 formation from treated EGFP or TXNIP transfected HEK cells. **c** Treatment of SMC with oxLDL significantly attenuates basal LC3 lipidation and leads to accumulation of LC3I. The graphs to the right show quantification of LC3I and LC3II from control, EBSS or oxLDL-treated SMC. **d** oxLDL induces Atg7 oxidation and significantly decreases the covalent interaction between Atg3 and LC3. The graphs to the right show quantification of Atg7 oxidation, Atg3-LC3 and Atg7-LC3 formation from control, EBSS or oxLDL-treated SMC. All data represent mean ± s.e. from 4−5 (**a**, **b**) or 3 independent experiments (**c**, **d**). *P < 0.05, **P < 0.01, ***P < 0.005 statistical significance (Dunnett’s test) relative to control or bafilomycin A1 alone. ††*P* < 0.01, †††*P* < 0.005 statistical significance relative to EBSS or EBSS + bafilomycin A1 treatment

Elevations in endogenous ROS formation compromises the cellular reducing system by oxidizing the constituent reducing enzymes^31,32^. Therefore, during conditions when oxidants are elevated, as commonly occurs during disease, we hypothesized that impairment of the reducing system could prevent the reduction of Atg3 and Atg7 required to maintain catalytic activity. To assess how loss in the reducing system during oxidative stress may impact on autophagy induction, we selectively attenuated thioredoxin activity by overexpressing its endogenous inhibitor, thioredoxin-interacting protein (TXNIP)^33^. The overexpression of TXNIP in HEK cells significantly attenuated LC3 lipidation in response to amino acid withdrawal, observed in the presence or absence of bafilomycin A1 (Fig. 6a). Despite this, TXNIP overexpression did not mitigate the loss in covalent interaction between LC3 and both Atg3 and Atg7 after amino acid starvation (Fig. 6b). Therefore, loss in LC3II formation is consistent with enhanced Atg3 and Atg7 oxidation that prevents interaction and lipidation of LC3.

**Fig. 7:**
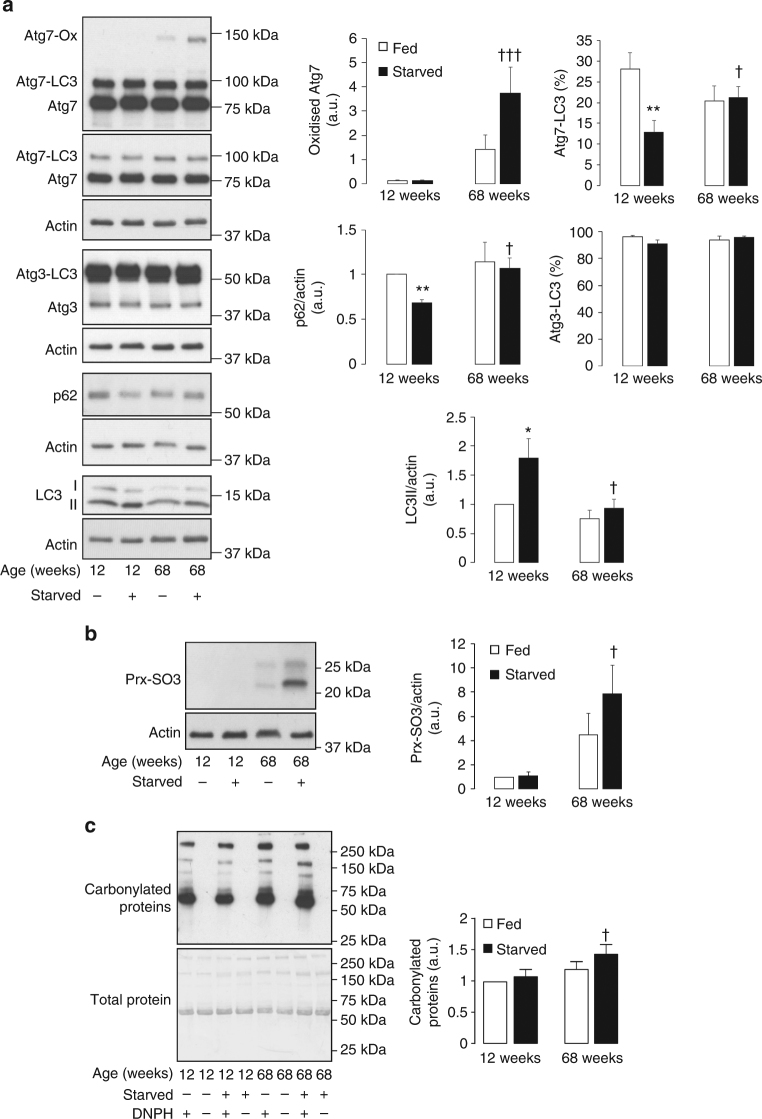
Loss in LC3 lipidation and increased Atg7 oxidation in aged mouse aorta. **a** Aged mice have impaired aortic LC3 lipidation, higher expression of p62, and loss of LC3-Atg7 in response to 24-h food withdrawal. In addition, aged mice had increased Atg7 oxidation in their aortic vessels, at a molecular weight consistent with a disulfide-bound Atg3 heterodimer. The graphs to the right show quantification of LC3 lipidation, p62, Atg7 oxidation, Atg3-LC3, and Atg7-LC3 formation in aorta from fed or starved young or aged mice. **b** The hyperoxidation of prx (Prx-SO_3_) was significantly enhanced in aortic vessels of aged mice after 24-h food withdrawal. The graph to the right shows quantification of Prx-SO_3_ in aorta from fed or starved young or aged mice. **c** Plasma protein carbonylation is significantly enhanced in aged starved mice. The graph to the right shows quantification of carbonylated plasma proteins from fed or starved young or aged mice. All data represent mean ± s.e. from 8–9 (**a**, **b**) mice/group except for analysis of p62 expression and plasma protein carbonylation (**c**) which was using five mice/group. *P < 0.05, **P < 0.01, statistical significance (Dunnett’s test) relative to young fed mice. †*P* < 0.05, †††*P* < 0.005 statistical significance relative to young starved mice

The ability of endogenous oxidants to inhibit LC3 lipidation was further substantiated using the pro-atherogenic lipoprotein oxLDL. When SMC were exposed to oxLDL, the basal lipidation of LC3 was inhibited (Fig. 6c). This loss in LC3 lipidation was due to increased Atg7 oxidation, which generated an oxidized complex at a molecular weight consistent with a disulfide-bound Atg3 heterodimer (Fig. 6d). The ability of oxLDL to induce detectable Atg7 oxidation without the need for amino acid deprivation was likely due to activation of the autophagy pathway. This is supported by a loss in Atg3 covalent interaction with LC3 upon treatment of SMC with oxLDL, consistent with upstream enzyme activation comparable to that of amino acid deprivation. As tissue aging is also associated with loss in cellular autophagy and elevated oxidant formation, we next assessed a potential role for Atg3 and Atg7 oxidation. In aged mice, the lipidation of aortic LC3 and a loss in p62 induced by 24-h food withdrawal was significantly impaired when compared to a younger cohort of mice (Fig. 7a). Consistent with this observation and enhanced oxidative stress, older mice had significantly increased disulfide oxidized Atg7, at a molecular weight consistent with a disulfide-bound Atg3 heterodimer. Furthermore, the presence of a more oxidizing environment within the aorta of aged mice was further substantiated by detection of hyperoxidized peroxiredoxin (prx), which was significantly higher after starvation (Fig. 7b)^31^. Also, consistent with an increase in oxidative stress in aged starved mice, carbonylation of plasma proteins was significantly enhanced (Fig. 7c). Therefore, an increased oxidizing environment within aged tissue leads to oxidation of Atg3 and Atg7, which decreases the ability to upregulate LC3 lipidation.

## Discussion

The discovery of a molecular mechanism for inhibiting autophagy induction by cellular oxidants is perhaps surprising, as oxidants are considered inducers of this catabolic process^34^. However, this is partially based on studies where activation of autophagy is observed after prolonged exogenous oxidant treatment, often for 24−48 h. After such prolonged treatment, it is likely that the initial oxidant will have been fully consumed by the replete cellular antioxidant and reducing systems. Therefore, acute inhibitory effects may be missed with observed increases in autophagy representing an adaptive cellular response. In addition, studies often do not consider the impact of autophagy flux on its sensitivity to regulation by ROS. This is because it is routine to monitor the impact of ROS on autophagy in cells where this process is unstimulated, therefore making an inhibitory effect difficult to observe. Furthermore, compartmentalized changes in ROS formation are likely to have disparate effects on autophagy. Certainly, there is evidence that physiological ROS formation can induce autophagy. This is through activation of select targets including ataxia-telangiectasia mutated and AMPK, by elevation in localized forms of ROS^35,36^. Whereas, under conditions of dysregulated oxidant formation or impairment in cellular reducing system, it is conceivable that oxidative modification of additional targets may prevent this catabolic process. Certainly, this is consistent with increased oxidants derived from nicotinamide adenine dinucleotide phosphate-oxidase isoform 2 (Nox2) in a mouse model of Duchenne muscular dystrophy^37^. In this mouse model, increased Nox2-derived oxidants led to impaired autophagy, which caused enhanced contractile dysfunction that is associated with the pathology of this muscular degenerative disease.

In this study, we used H_2_O_2_ to mimic oxidative stress with concentrations selected based on previous studies and suitability for mimicking pathological conditions^28,38,39^. Here we found increased lipidation of LC3 by amino acid deprivation was robustly attenuated in HEK and SMC by exogenous H_2_O_2_ treatment, which was due to direct enzyme oxidation independent of protein expression. Loss in LC3 lipidation prevented its transport to autophagic vacuole membranes, where it is known to play an important role in autophagosome maturation^7–10^. The inhibition of LC3 lipidation in HEK cells required a higher dose of H_2_O_2_ compared to SMC, perhaps reflecting a more reductive environment within the kidney cells^40^. However, in SMC a concentration of 100 μm H_2_O_2_ was sufficient in attenuating the lipidation of LC3 during an hour of amino acid deprivation. The ability of H_2_O_2_ to also inhibit tat-beclin1-induced LC3 lipidation allowed us to narrow down the target(s) of oxidation responsible for inhibiting LC3 lipidation, by focusing on those downstream of beclin1^23^. This led us to speculate that the targets of oxidation were Atg3 and Atg7. The rationale being Atg3 and Atg7 have surface accessible catalytic cysteine thiols that bind to LC3. Therefore, oxidation of these cysteines would logically prevent LC3 interaction and PE conjugation. On analyzing Atg3 and Atg7 under non-reducing conditions we observed stable covalent complexes with LC3 under fed conditions. The ability of beta-mercaptoethanol to cleave the thio-ester bond linking LC3 to both Atg3 and Atg7 likely explains why stable covalent complexes have not been previously reported in cultured mammalian cells or tissue, as it is routine to resolve samples under reducing conditions. The induction of autophagy by amino acid withdrawal decreased the stable covalent interaction between LC3 and Atg7 in HEK cells, and between LC3 and both Atg3 and Atg7 in SMC. A rational explanation for this loss in stable covalent interaction is due to increased conjugase activity, where LC3 is actively transferred between active site thiols on Atg7 to Atg3 then attached to PE before being released. Activation of Atg3 is stimulated by its membrane-curvature-sensing domain upon formation of autophagosomal lipid bilayers during amino acid withdrawal^6^. Therefore, loss in detectable LC3 covalent interaction provides a measure of autophagy induction, as it directly correlates with upstream activation of Atg3 and Atg7. The loss in covalent LC3 interaction with Atg3 and Atg7 during amino acid withdrawal sensitized these enzymes to oxidant-dependent intermolecular disulfide bond formation within SMC. This is consistent with oxidation of the catalytic cysteine thiols, which are more accessible during autophagy activation, due to loss in covalent-bound LC3. This is also consistent with the crystal structure of yeast Atg3 and Atg7, where each enzyme has a conserved catalytic site that is closely aligned, allowing transfer of LC3 but also enough proximity for intermolecular disulfide formation (Fig. 2e)^27^. In HEK cells there was a lack of intermolecular disulfide detection, which could be explained by the enhanced occupancy of the catalytic site on Atg3 with LC3 during amino acid withdrawal compared to SMC, which is 10.5% higher. This increase in bound LC3 on Atg3 diminishes the likelihood of both enzymes in a complex having catalytic cysteines free to form a disulfide. However, if either Atg3 or Atg7 alone have an unoccupied catalytic cysteine within the same complex then reversible thiol oxidation can occur, which was detected using the PEG-switch assay, and would likely result in S-glutathiolation. As well as Atg3 and Atg7 oxidation, we detected reversible thiol modification of cellular Atg4B and Atg10 by H_2_O_2_. These enzymes contain catalytic thiols, with Atg4B having been previously described as a target of oxidation^29^. However, the oxidation of Atg10 did not impact on conjugation of Atg5 to Atg12 needed for LC3 lipidation^4^. In addition, loss of LC3 lipidation by H_2_O_2_ was found to be independent of Atg4B. It is perhaps unsurprising that the oxidation of Atg10 and Atg4B is not responsible for the rapid loss in LC3 lipidation observed in cells exposed to H_2_O_2_, as these enzymes are constitutively active. As Atg5 and Atg12 are fully conjugated and LC3 already cleaved, Atg10 or Atg4 would have to remain inhibited by thiol oxidation over an extensive period (requiring de novo protein synthesis) to impact on LC3 lipidation, which would already be impaired by Atg3 and Atg7 oxidation. However, it cannot be ruled out that regulators of autophagosome formation upstream of Atg3 and Atg7 may also undergo redox regulation that impacts on the acute regulation of autophagy.

**Fig. 8:**
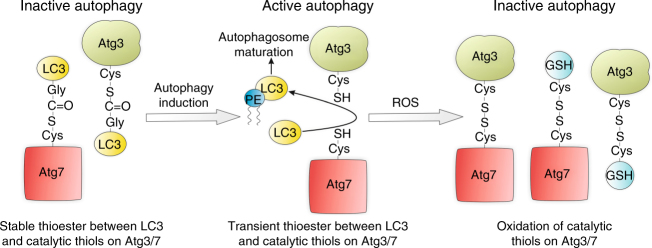
Model for the inhibition of autophagy by Atg3 and Atg7 oxidation. LC3 forms stable covalent complexes with inactive Atg3 and Atg7. When autophagy is stimulated stable covalent interactions between LC3 and both Atg3 and Atg7 become transient, due to enzyme activation. Loss in stable covalent LC3 interaction increases the catalytic thiol availability of Atg3 and Atg7 to direct oxidation by cellular oxidants. If catalytic thiols are available on both Atg3 and Atg7 within the same complex then intermolecular disulfide formation can occur under oxidizing conditions. However, if only a single catalytic thiol is available within the Atg3/ Atg7 complex, then oxidation will likely lead to stable S-glutathiolation (GSH). The oxidation of catalytic thiols on Atg3 and Atg7 prevents lipidation of LC3 required for autophagosome maturation, thus inhibiting cellular autophagy

The reversible oxidation of Atg3 and Atg7 detected using the PEG-switch assay is likely S-glutathiolation due to the high cellular abundance of GSH. This is consistent with the direct reversible inhibition of the highly homologous ubiquitin-activating enzyme (E1) and ubiquitin-conjugating enzymes (E2) through oxidative S-glutathiolation of their catalytic thiols^41^. Furthermore, this would mean the oxidant-induced intermolecular disulfide between Atg3 and Atg7 observed in SMC is likely to be an underestimate of total reversible inhibitory oxidation. This is due to additional S-glutathiolation of catalytic cysteines on either Atg3 or Atg7 when the adjacent enzyme in the complex is bound to LC3 (Fig. 8). In addition, as autophagy activation is required for Atg3 and Atg7 oxidation, this explains why inhibition of this catabolic process by ROS has not been widely observed. This is because the impact of oxidants on autophagy is routinely analyzed in cells where this process is unstimulated, thus limiting the ability of Atg3 and Atg7 to undergo oxidation.

Excessive autophagy can drive cell death through a process termed autosis^42^. It is therefore conceivable that oxidant-dependent inhibition of Atg3 or Atg7 could preserve cell integrity when this catabolic process is overly stimulated. However, extended treatment with H_2_O_2_ is likely to compromise cell integrity through activation of apoptopic or necrotic pathways^43^. Therefore, development of therapies that selectively target thiols on Atg3 or Atg7 may provide a mechanism for preventing autosis, without the risk of compromising cell integrity.

As well as LC3, its paralogs GABARAPL1 and 2 are substrates for Atg3 and Atg7, were also found to form stable covalent complexes within cultured cells. The molecular weights of these complexes were consistent with covalent bound Atg3 or Atg7. During autophagy the lipidation of GABARAPL1 and 2 is crucial in the late stage of autophagosome biogenesis, as it is required for sealing the isolation membrane^8^. Therefore, it is likely that oxidation of Atg3 and Atg7 will also prevent the lipidation of GABARAPL1 and 2, enzymes that are essential for autophagosome maturation.

The interaction of Atg3 and Atg7 required for transfer and lipidation of LC3 was found to be inhibited by enzyme oxidation^27^. As the interaction between Atg3 and Atg7 was only disrupted by H_2_O_2_ when cells were deprived of amino acids, this suggests that oxidation of the active site residue(s) is responsible for this observation. This is likely due to the addition of a bulky glutathione moiety to the enzymes active site, which either directly or through a conformational change prevents enzyme interaction. Furthermore, this highlights the likely underestimation of intermolecular disulfide bond formation between Atg3 and Atg7 as a measure of total inhibitory enzyme oxidation.

In an in vitro lipidation assay, oxidized glutathione prevented LC3II formation that was consistent with Atg3 and Atg7 catalytic thiol oxidation through S-glutathiolation. This substantiated findings from cell studies, as oxidation of Atg3 and Atg7 alone is sufficient in preventing LC3 lipidation. In addition, in vitro lipidation assays confirmed the formation of stable covalent complexes between LC3 and both Atg3 and Atg7. Furthermore, in an attempt to mitigate the effects of H_2_O_2_ on LC3 lipidation, we increased the intracellular pool of reduced Atg7. Here overexpression of Atg7 improved LC3 lipidation in amino-acid-starved cells exposed to H_2_O_2_. This finding further substantiated the importance of Atg7 oxidation in mediating the inhibition of autophagy, but also provides a potential strategy to improve autophagy flux under conditions of oxidative stress. In addition, the formation of a disulfide-bound Atg3 and Atg7 heterodimer under oxidizing conditions was confirmed, as siRNA knock-down of either enzyme attenuated covalent complex formation. Here the catalytic thiol of Atg3 and Atg7 was identified as the site of oxidation, as mutation of the active site cysteine on either enzyme prevented disulfide-dependent heterodimerization.

The impact of endogenous oxidant formation on acute autophagy induction was also investigated by attenuating the cellular thioredoxin reducing system, using overexpression of the endogenous thioredoxin inhibitor TXNIP^33^. Inhibiting the cellular thioredoxin reducing system significantly attenuated lipidation of LC3 induced by amino acid withdrawal. In addition, TXNIP overexpression did not reverse the loss in covalent interaction between LC3 and both Atg3 and Atg7 after amino acid withdrawal. Consequently, inhibiting the cellular thioredoxin reducing system did not appear to impact on the upstream activation of Atg3 and Atg7 during amino acid starvation, consistent with normal autophagosome nucleation. Therefore, loss in LC3 lipidation when inhibiting thioredoxin is likely through enhanced inhibitory Atg3 and Atg7 catalytic thiol oxidation. Therefore, these findings provide evidence that an active cellular reducing system is required to maintain functional autophagy, likely by keeping the active site thiols of Atg3 and Atg7 reduced to preserve their ability to interact with LC3. To further assess the role of intracellular oxidant formation on autophagy induction we treated aortic SMC with oxLDL. The rational for selecting oxLDL was its relevance to aortic SMC pathophysiology, which is associated with elevated endogenous oxidant formation. Certainly, oxLDL has been implicated in the development of atherosclerotic plaque formation, which is attributed in part to increased vascular smooth muscle cell (VSMC) death. This ability of oxLDL to induce VSMC apoptosis is dependent on ROS generated by lipoxygenase and mitochondrial pathways^44,45^. Treatment of amino-acid-fed SMC with oxLDL prevented the lipidation of LC3. This observation was consistent with the ability of oxLDL to increase ROS, confirmed by elevated disulfide oxidation of Atg7, which generated a molecular weight complex corresponding to bound Atg3. The ability of oxLDL to induce detectable Atg7 oxidation in fed cells is likely due to its ability to stimulate the autophagy pathway, observed by a loss in covalent LC3 bound to Atg3 that is comparable to amino acid deprivation. Activation of the autophagy pathway by oxLDL would decrease covalent-bound LC3, providing increased access of the catalytic thiol on Atg3 to disulfide oxidation. With a concomitant increase in oxidant generation, this could explain how oxLDL promotes formation of an oxidized higher molecular weight complex for Atg7 and prevents LC3 lipidation.

To fully validate the importance of Atg3 and Atg7 oxidation in regulating autophagy, this process was assessed in a relevant in vivo model. We chose to use a mouse model of aging, as this process is causatively linked to a loss in autophagy flux and enhanced oxidant formation^14,21^. Following our observations in cultured aortic SMC we assessed autophagy flux in the aorta of fed and food-deprived mice at 12 and 68 weeks of age. In food-deprived mice the lipidation of LC3 was impaired and abundance of p62 increased in aorta of aged compared to young mice, consistent with dysfunctional autophagy induction. This impairment in autophagy correlated with an increase in inhibitory Atg7 oxidation in aged mouse aorta, which generated a molecular weight complex consistent with disulfide-bound Atg3. The increased oxidizing environment in aged mouse aorta that accounted for Atg7 oxidation was further supported by increased prx hyperoxidation and plasma protein carbonylation, which significantly increased in aged mice after 24-h food withdrawal^31^.

To conclude we have identified a process for the thiol-dependent regulation of autophagy, through oxidation of Atg3 and Atg7, which prevents PE conjugation to LC3 required for autophagosome maturation. Stable covalent complexes identified between LC3 and both Atg3 and Atg7 protects each enzyme from inhibitory oxidation. However, under conditions where autophagy is stimulated, loss in stable LC3 interaction enhances the susceptibility of Atg3 and Atg7 to direct oxidative inactivation. This mechanism is likely to play an important role in regulating autophagy, including its loss during aging, as well as providing therapeutic targets to regulate autophagosome maturation.

## Methods

### Cell culture

SMC were isolated from explanted rat aorta. In brief, isolated rat aorta was cleaned of fat and connective tissue, opened lengthways and cut into sections. Sections of aorta were placed lumen side down in tissue culture plates and covered with media (Dulbecco’s modified eagle medium ((DMEM) supplemented with 10% fetal calf serum (FCS) and 1% pen/strep) to encourage SMC growth onto the plate surface. Once SMC had grown and adhered onto the plate, the aortic sections were removed, with cells then maintained in culture media. SMC and HEK (240073, stratagene) cells were cultured in DMEM (GIBCO, Life Technologies) supplemented with 10% FCS and 1% penicillin/streptomycin and kept at 37 °C in an incubator with 5% CO_2_. To induce autophagy cells were rinsed and then incubated with Earle’s balanced salt solution (EBSS) for 1 h. At the initiation of starvation some cells were exposed to H_2_O_2_ and/or 100 nm bafilomcyin A1. In some experiments HEK cells were transfected for 24 h with 100 ng of EGFP (Addgene, gift from Benjamin Glick, 18758), 100 ng of EGFP-TXNIP (Addgene, gift from Clark Distelhorst, 36412), 10 ng of Atg7 (Sino Biological, HG15684-UT), 10 ng pCMV-myc-LC3 (Addgene, gift from Toren Finkel, 24919) or 10 ng pCMV-myc-LC3 ΔC DNA using lipofectamine 2000 (Invitrogen) before treatment^46,47^. The pCMV-myc-LC3 ΔC was generated to express a truncated form of human LC3 without the C-terminal 5 amino acids (MKLSV) normally cleaved by Atg4. pCMV-myc-LC3 ΔC was generated from wild-type by introducing a premature stop codon using site directed mutagenesis (QuikChange II XL, Agilent) following the manufacturer’s instructions. In some experiments, SMC were transfected for 48 h with a combination of 250 ng of wild-type mCherryAtg3 (Addgene, 54993), C264A mCherryAtg3, wild-type Atg7 (Sino Biological, HG15684) or C572A Atg7 using lipofectamine 3000. In other experiments SMC were treated with human oxLDL (Alfa Aesar) in complete growth media for the indicated time. To inhibit protein synthesis SMC were preincubated for 1 h with 100 μg/ml cycloheximide before a further 1 h treatment with bafilomycin in combination with or without EBSS or H_2_O_2_. In experiments assessing cell viability, after treatment cells were detached using trypsin-EDTA (ThermoFisher), diluted into trypan blue, and then percentage of stained cells analyzed using an automated cell counter (TC20, Bio-Rad). When assessing cellular caspase-3 cleavage a positive control was generated by treating cells for 6 h with 3 μm staurosporine (Sigma), and for assessing proteasomal degradation SMC were pretreated for 30 min with 10 μm MG132, then co-treated with experimental reagents for 1 h. To knock-down expression of Atg3 or Atg7 SMC were transfected with siRNA (ON-TARGETplus SMARTpool siRNA, Dharmacon) using Lipofectamine 3000, following the manufacturer’s instructions. Forty-eight hours after transfection cells were left in complete media or amino acid starved in the presence or absence of H_2_O_2_ for 1 h, before lysis into non-denaturing sample buffer containing maleimide. To monitor intracellular ROS SMC or HEK cells seeded on 96-well plates were pre-treated with 5 µm 6-carboxy-2′,7′-dichlorodihydrofluorescein diacetate (carboxy-H_2_DCFDA, ThermoFisher), in DMEM containing 2% FCS for 30 min in a 37 °C culture incubator. After incubation media was exchanged with control media or autophagy stimulated (EBSS or tat-belcin1) in the presence or absence of H_2_O_2_. Cells were then maintained at 37 °C for 1–3 h before being assessed for carboxy-H_2_DCFDA fluorescence (λex 595 nm; λem 520 nm) using a plate reader (SpectraMax GeminiXS, Molecular Devices).

### Quantitative PCR analysis

mRNA from SMC that were fed, amino acid starved or amino acid starved and H_2_O_2_ treated for 1 h was isolated and reverse transcribed using a TaqMan gene expression cells-to-CT kit (ThermoFisher). Quantitative PCR was performed using TaqMan gene expression assays and a StepOnePlus Real-Time PCR System using the manufacturer’s instructions. Samples were assessed for LC3A (Rn00458724_g1, ThermoFisher) and LC3B (Rn00575883_m1, ThermoFisher) gene expression, as well as β-actin (Rn00667869_m1, ThermoFisher) as an internal control. Although LC3A transcripts could be detected, due to the low expression of LC3B in SMC, we were unable to reliably measure its gene expression.

### Autophagy induction using tat-beclin1

A cell-permeable autophagy inducing peptide (Tat-beclin1, Eurogentec) consisting of the autophagy essential residues of beclin1 and the HIV virus Tat sequence was used to induce autophagy. The peptide (50 µm) was administered to cells as previously described for 3 h in OPTIMEM acidified with 15% (vol/vol) 6 n HCl^23^. In some experimental groups 100 nm bafilomcyin A1 was also included, and 1 h after incubation some groups were exposed to H_2_O_2_.

### Immunoblotting

After incubation cells were lysed directly into sample buffer (50 mm Tris-HCl buffer pH 6.8, 2% SDS, 10% glycerol, 0.005% bromophenol blue) containing 100 mm maleimide to preserve protein oxidation. Protein samples were resolved on SDS-PAGE gels and then transferred to polyvinylidene difluoride membranes by western blotting (TransBlot Turbo, Bio-Rad). Membranes were blocked in milk dissolved in phosphate-buffered saline (PBS) supplemented with 1% Tween before incubation with primary antibodies. Membranes were developed using enhanced chemiluminescence substrate (ThermoFisher) and detected using photographic film, which was developed using an automatic processor (Fuji RG II). Samples were immunoblotted for LC3 (1:1000, Cell Signaling), Atg3 (1:5000, Abcam), Atg7 (1:1000, Cell Signaling), Actin (1:10,000, Sigma), Atg5 (1:1000, Cell Signaling), Atg12 (1:1000, Cell Signaling), Atg4B (1:1000, Cell Signaling), Atg10 (1:1000, Abcam), GABARAPL1 (1:1000, Cell Signaling), GABARAPL2 (1:1000, Cell Signaling), cGMP-dependent protein kinase (PKG, 1:1000, ENZO Life Sciences), Myc-tag (1:1000, Cell Signaling), p62 (1:500, Cell Signaling), caspase-3 (1:1000, Abcam), peroxiredoxin-SO3 (1:500, AbFrontier) and GFP (1:2000, Miltenyi Biotec). All uncropped western blots can be found in Supplementary Fig. 6.

### Immunoprecipitation

HEK cells were mock or myc-LC3 DNA transfected for 24 h and then either left in complete media or amino acid starved for 1 h. Alternatively, SMC were transfected with Atg7 and mCherry-Atg3 for 48 h before being left untreated or amino acid starved for 1 h with 100 μm H_2_O_2_ co-treatment. After incubation cells were rinsed in PBS and then lysed into ice-cold PBS containing 1% Triton X-100 and protease inhibitors (cOmplete, Roche). Cell lysates were then centrifuged at 250,000 r.p.m. for 5 min at 4 °C to pellet insoluble material. Supernatants were added to magnetic beads conjugated to myc-tag antibody (Cell Signaling), with some also added to sample buffer for immunoblot analysis. After 24 h incubation at 4 °C, beads were washed five times with PBS containing 1% Triton-X100. Bound proteins were then eluted by addition of sample buffer and assessed by immunoblot analysis.

### Electron microscopy analysis

SMC grown on coverslips were left untreated or placed into EBSS for 1 h with or without the addition of H_2_O_2_. After incubation cells were washed three times with PBS then fixed for 1 h in 4% (wt/vol) paraformaldehyde before being permeabilized for 15 min using 40 μg/ml digitonin. Permeabilized cells were then blocked for 30 min in 1% (wt/vol) bovine serum albumin (BSA) solubilized in PBS before being immuno-labeled with an anti-LC3 antibody (CAC-CTB-LC3–2-IC, Cosmo Bio) diluted to 1:80 in the same buffer. After 2 h incubation cells were washed with PBS and then incubated for a further 2 h with secondary anti-mouse nano-gold (Universal Biologicals) diluted to 1:200 in 1% (wt/vol) BSA solubilized in PBS. After incubation cells were then fixed for a second time using 2% (wt/vol) PFA/2% (vol/vol) glutaraldehyde in 0.15 m cacodylate buffer for 1 h. To increase the size of nano gold particles, cells were subsequently incubated with GoldEnhance EM Plus (Nanoprobes) for 15 min in accordance with the manufacturer’s instructions. The cells were then incubated in 1% (vol/vol) osmium tetroxide/1% (wt/vol) potassium ferrocyanide for 1 h followed by 1% (wt/vol) uranyl acetate for an additional hour. Using increasing concentrations of ethanol followed by propylene oxide, the cells were embedded in epon resin. Sections (~100 nm) were cut and images were acquired on a JEOL 1010 transmission electron microscope. Autophagic vacuoles were identified by the presence of membrane like-structures with LC3 immuno-gold labeling.

### PEG-switch assay

Reversible protein oxidation was assessed as previously described using the PEG-switch assay^48^. In brief untreated cells or those starved for 1 h in EBSS with or without H_2_O_2_ co-treatment were lysed into alkylating buffer (1% SDS, 100 mm maleimide, 100 mm Tris-HCl pH 7.4) and then heated at 50 °C for 25 min. Alkylated samples were then supplemented with 200 mm dithiothreitol (DTT) to quench the maleimide and reduce reversibly oxidized thiols. After 30 min incubation, each sample was desalted (Zeba columns, Peirce) and eluted into labeling buffer (10 mm PEG-maleimide, 1% SDS and 100 mm Tris-HCl pH 7.4). Following 2 h further incubation at room temperature samples were prepared for SDS-PAGE by addition of sample buffer containing 5% beta-mecaptoethanol and immunoblotted as described above.

### LC3 lipidation assay

Phospholipids were purchased in chloroform. 2-oleoyl-1-palmitoyl-sn-glycero-3-phosphocholine, 1,2-dioleoyl-sn-glycero-3-phosphoethanolamine and 1,2-dioleoyl-sn-glycero-3-phospho-l-serine (Avanti Polar Lipids) were mixed in a 1:1:1 molar ratio in glass tubes and dried in a vacuum centrifuge to remove any chloroform to produce a dried lipid film. Dried lipids were reconstituted to a final concentration of 1 mm in buffer (25 mm Tris-HCl pH 7.5, 137 mm NaCl, 2.7 mm KCl). The lipid mixture was then vortexed vigorously and sonicated on ice for 2 min to create unilamellar vesicles (liposomes). For the lipidation of LC3, 30 µm liposome were mixed with 5 µm recombinant human His-MAP1LC3B (Fitzgerald Industries), 1 µm of recombinant human His6-Atg3 and His6-Atg7 (R&D systems), 0.2 mm DTT and 10 mm MgCl2 in reaction buffer (50 mm Tris-HCl pH 7, 150 mm NaCl). Reactions were initiated by addition of 5 mm ATP and incubated at 30 °C for up to 60 min before being terminated by the addition of sample buffer containing either 5% 2-mecaptoethanol or 100 mm maleimide. Samples were then resolved on SDS-PAGE gels and proteins stained using colloidal Coomassie.

### Confocal microscopy

Proximity ligation assay (PLA) was performed using the Duolink In Situ kit (Sigma) following the manufacturer’s instructions. In brief, SMC were left untreated or treated with EBSS with or without 100 µm H_2_O_2_ for 1 h then fixed in 4% paraformaldehyde for 10 min. After washing in PBS, cells were permeabilized for 5 min in 0.2% Triton X-100. Cells were blocked for 1 h at 37 °C then incubated overnight with primary Atg3 and Atg7 antibodies (Abcam) at 1:50 dilution. After washing to remove primary antibodies, Duolink probes were added and cells were incubated at 37 °C for a further hour. Duolink Ligation solution was prepared as described with the supplied ligase, with the reaction allowed to proceed for 30 min at 37 °C. Duolink Amplification solution was prepared as described and polymerization proceeded for 100 min at 37 °C. Finally, slides were prepared for confocal microscopy by adding coverslips with mounting media containing 4',6-diamidino-2-phenylindole (DAPI). In separate experiments, LC3 localization was assessed in fed or amino-acid-starved SMC after 1 h with or without H_2_O_2_ co-treatment. These cells were fixed and permeabilized as with the PLA, then incubated with LC3 antibody (1:50, Cell Signaling) overnight at 4 °C. After washing to remove primary antibody, Cy3 anti-rabbit secondary antibody (1:100, Jackson ImmunoResearch) was added for 1 h at room temperature. After incubation cells were washed and then coverslips added with mounting media containing DAPI. All samples were analyzed using a Leica SP5 confocal microscope.

### Isolation of mouse aorta

Mice were maintained in accordance with the Home Office Guidance. Male C57BL/6J mice at 12-weeks and 68-weeks of age were purchased from Charles River. A cohort of young and aged mice were either kept on a standard RM1 diet or deprived of food for 24 h. After 24 h each mouse was anaesthetized and aorta carefully excised, cleaned of fatty tissue then rinsed in sterile PBS. Once cleaned, vessels were snap frozen in liquid nitrogen. Frozen vessels were then crushed under liquid nitrogen and placed into 5% wt/vol sample buffer containing 100 mm maleimide. Proteins were analyzed by immunoblotting as described above.

### Detecting of plasma protein carbonylation

Whole blood was drawn from the vena cava in the presence of heparin to prevent clotting, stored on ice and centrifuged at 1000×*g* for 10 min at 4 °C. The upper separated layer of plasma, free of red blood cells, was snap frozen in liquid nitrogen. Protein carbonyl content of plasma samples was analyzed using an OxyBlot protein oxidation detection Kit (Millipore). Protein (10  µg) from each plasma sample was denatured in 6% SDS in duplicate; these were then subject to derivatization with DNPH solution or derivatization-control solution for 15 min at room temperature. Samples were then immunoblotted as described above using a DNP antibody (1:150) provided with the OxyBlot kit. Total protein labeling in each lane was analyzed, with the band observed at 60 kDa omitted from analysis, as labeling of this protein could not be reliability assessed due to the extent of its modification by DNP.

### Statistical calculations

Results are presented as mean ± standard error of the mean (s.e.m.). Differences between groups were assessed using analysis of variance followed by a two-tailed unpaired Student's *t*-test or for multiple comparisons a Dunnett post-hoc analysis. Results were considered significant at the 95% confidence level.

### Data availability

The authors declare that all data supporting the findings of this study are available within the article and its supplementary information files or from the corresponding author upon reasonable request.

## Electronic supplementary material

Supplementary Information
