# Supplementary material for: Oxidation of Atg3 and Atg7 mediates inhibition of autophagy
Source: Nat Commun. 2018 Jan 8;9:95. doi: 10.1038/s41467-017-02352-z (PMC5758830; doi:10.1038/s41467-017-02352-z)

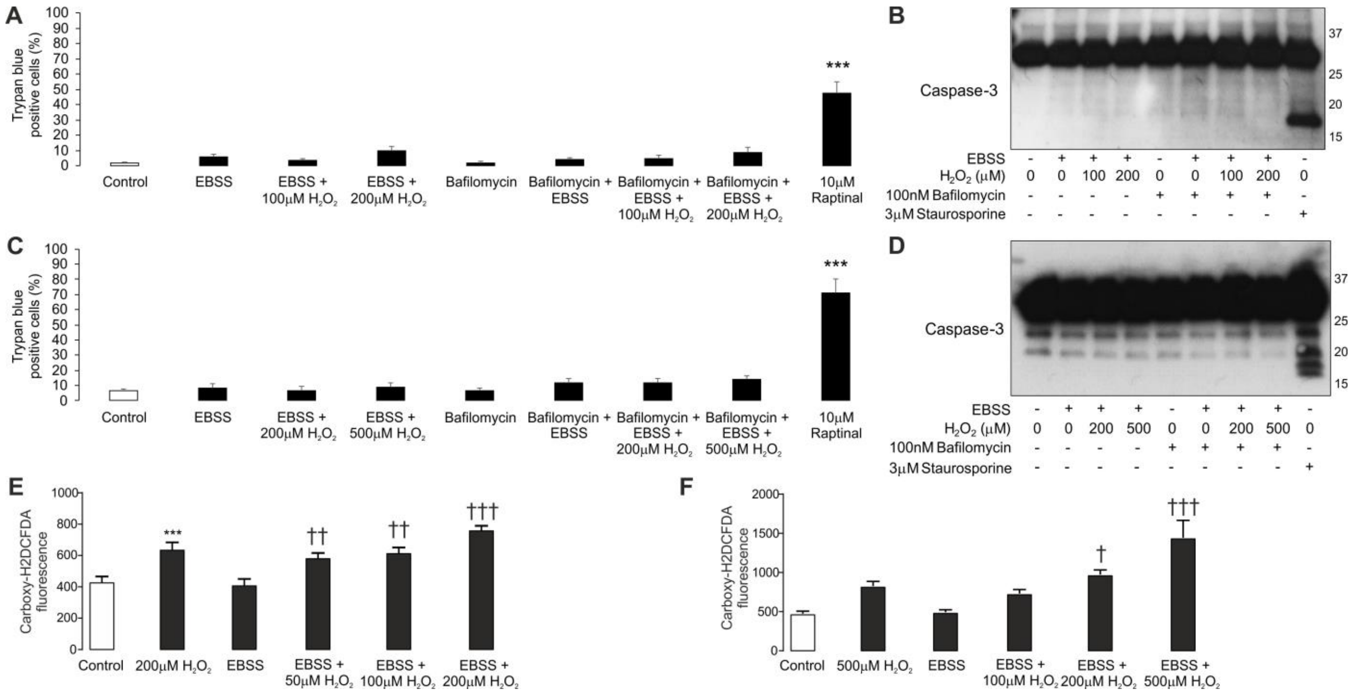

Supplementary Figure 1 Increased oxidative stress but maintained viability of cells exposed to H_2O_2. (A) Treatment of SMC with EBSS in combination with H_2O_2 or bafilomycin over 1 hour does not significantly impact cell viability, which is decreased by the rapid inducer of apoptosis raptinal. (B) Caspase-3 is not cleaved in amino acid starved SMC co-treated with H_2O_2 in the presence or absence of bafilomycin, but is enhanced by staurosporine. (C) Treatment of HEK cells with EBSS in combination with H_2O_2 or bafilomycin over 1 hour also does not significantly induce cell death, which is enhanced by raptinal. (D) Caspase-3 is not cleaved in amino acid starved HEK cells co-treated with H_2O_2 in the presence or absence of bafilomycin, but is enhanced by staurosporine. (E) SMC intracellular ROS was not enhanced over 1 hour by amino acid starvation, but was increased by exogenous H_2O_2 treatment. (F) In HEK cells amino acid starvation also did not increase ROS formation, which was enhanced by the addition of H_2O_2. All data represent mean ± s.e. from 3-4 (A, C) or 5 (E,F) independent experiments. ***$P < 0.005$ statistical significance (Dunnett's test) relative to control. †$P < 0.05$, ††$P < 0.01$, †††$P < 0.005$ statistical significance relative to EBSS treatment.

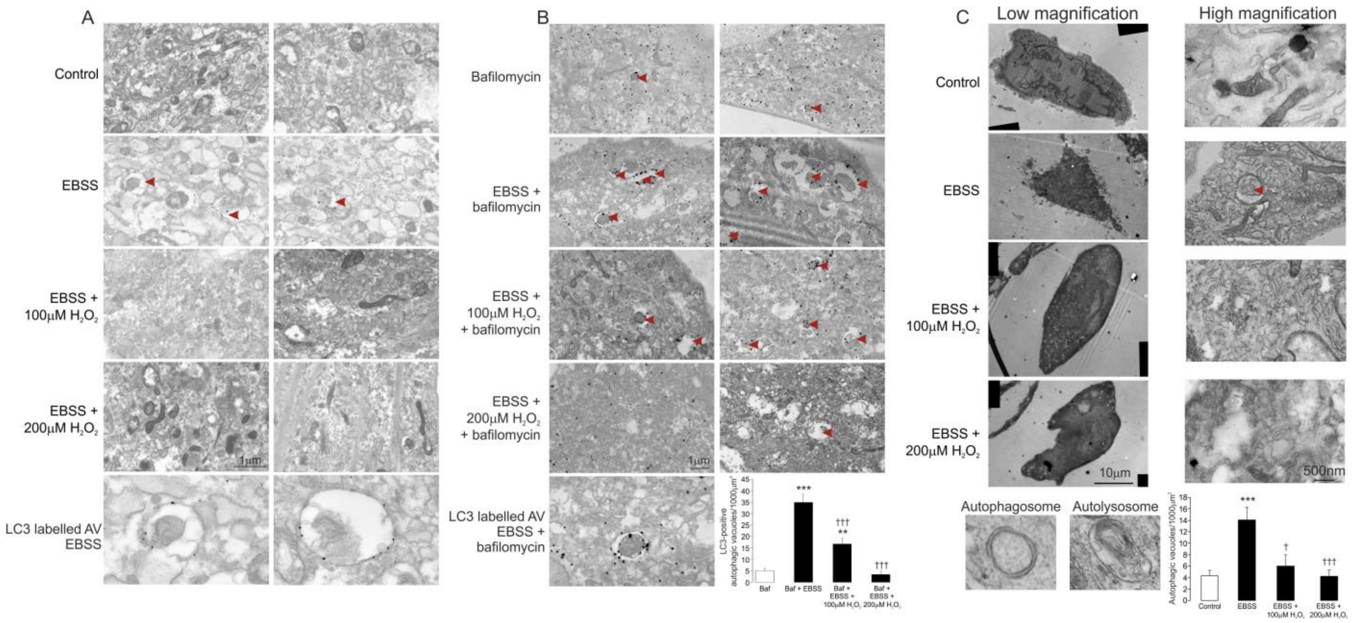

Supplementary Figure 2 Loss in LC3-positive autophagic vacuoles in SMCs exposed to H_2O_2. (A) Electron microscopy of immunogold-labelled LC3 in SMC shows increased LC3 positive autophagic vacuoles (arrows) following amino acid starvation that is lost in cells exposed to H_2O_2. (B) In the presence of bafilomycin amino acid starvation increased LC3 immunogold labelling of autophagic vacuoles (arrows) in SMC, which is attenuated by H_2O_2 (C) Total autophagic vacuoles (arrow) increase with amino acid deprivation, which is attenuated by H_2O_2 treatment. Also shown are examples of an autophagosome and autolysosome identified in amino acid starved SMC. All data represent mean ± s.e. from 5 (B, C) imaged cells. **$P < 0.005$, ***$P < 0.005$ statistical significance relative to control of bafilomycin treatment. †$P < 0.05$, †††$P < 0.005$ statistical significance relative to EBSS or EBSS + bafilomycin treatment.

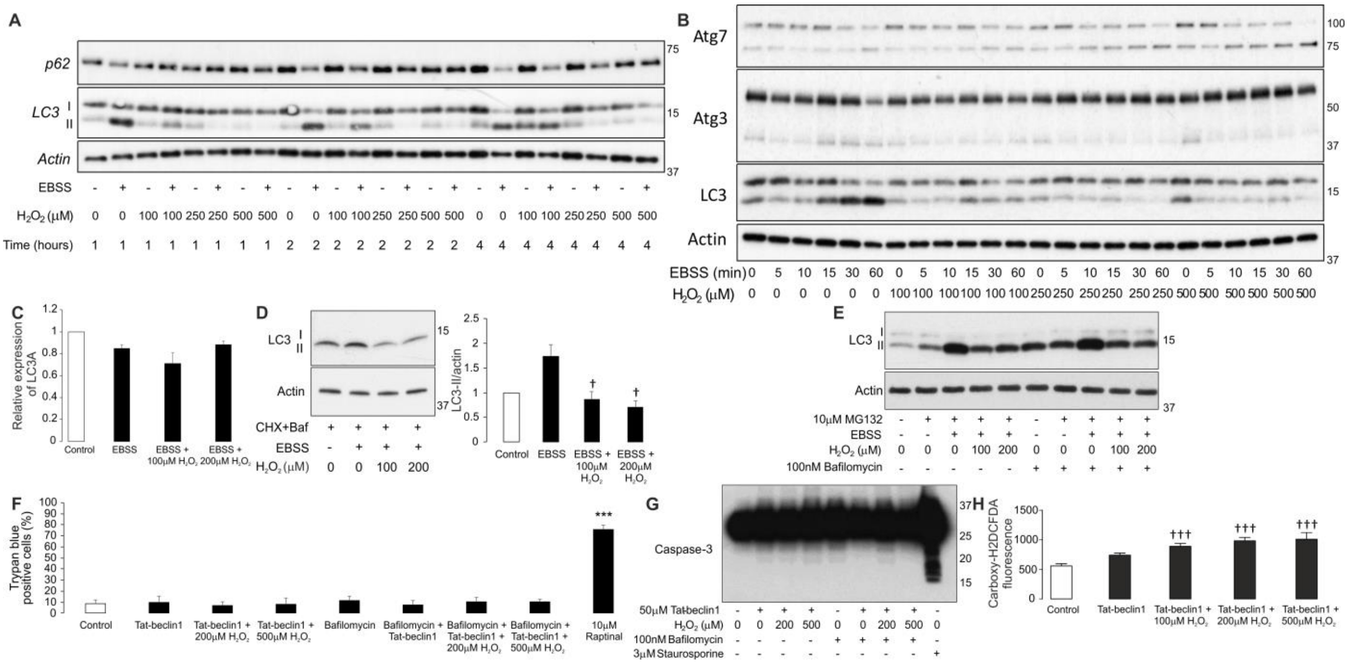

Supplementary Figure 3 Rapid and sustained inhibition of LC3 lipidation by H_2O_2. (A) The ability of H_2O_2 to inhibit LC3 lipidation and p62 degradation after amino acid withdrawal is sustained over 4 hours. (B) lipidation of LC3 occurs rapidly after amino acid withdrawal within 15 to 30 minutes and is inhibited by H_2O_2 treatment. In addition, treatment with H_2O_2 enhances the rate at which stable interaction between LC3 and Atg7 is lost, consistent with enzyme oxidation. (C) The expression of SMC LC3A is not significantly impacted by 1 hour H_2O_2 treatment. (D) pre-incubation of SMC with cycloheximide does not prevent loss in LC3 lipidation in amino acid deprived cells exposed to H_2O_2. The graph to the right shows quantification of LC3 lipidation in cycloheximide treated SMC. (E) Proteasome inhibition does not prevent loss in LC3 lipidation in amino acid starved SMC co-treated with H_2O_2, in the presence or absence of bafilomycin. (F) Treatment of HEK cells with tat-beclin1 in combination with H_2O_2 in the presence or absence of bafilomycin does not significantly impact cell viability, which is decreased by 1 hour raptinal treatment. (G) Caspase-3 is not cleaved in tat-beclin1 treated HEK cells co-treated with H_2O_2 in the presence or absence of bafilomycin, but is enhanced by staurosporine. (H) In HEK cells tat-beclin1 treatment did not significantly impact on ROS formation, which was enhanced by co-treatment with H_2O_2. All data represent mean ± s.e. from 4 (C), 3 (D), 5-6 (F), 9 (H) independent experiments. ***$P < 0.005$ statistical significance relative to control. †$P < 0.05$, †††$P < 0.005$ statistical significance relative to EBSS or tat-beclin1 treatment.

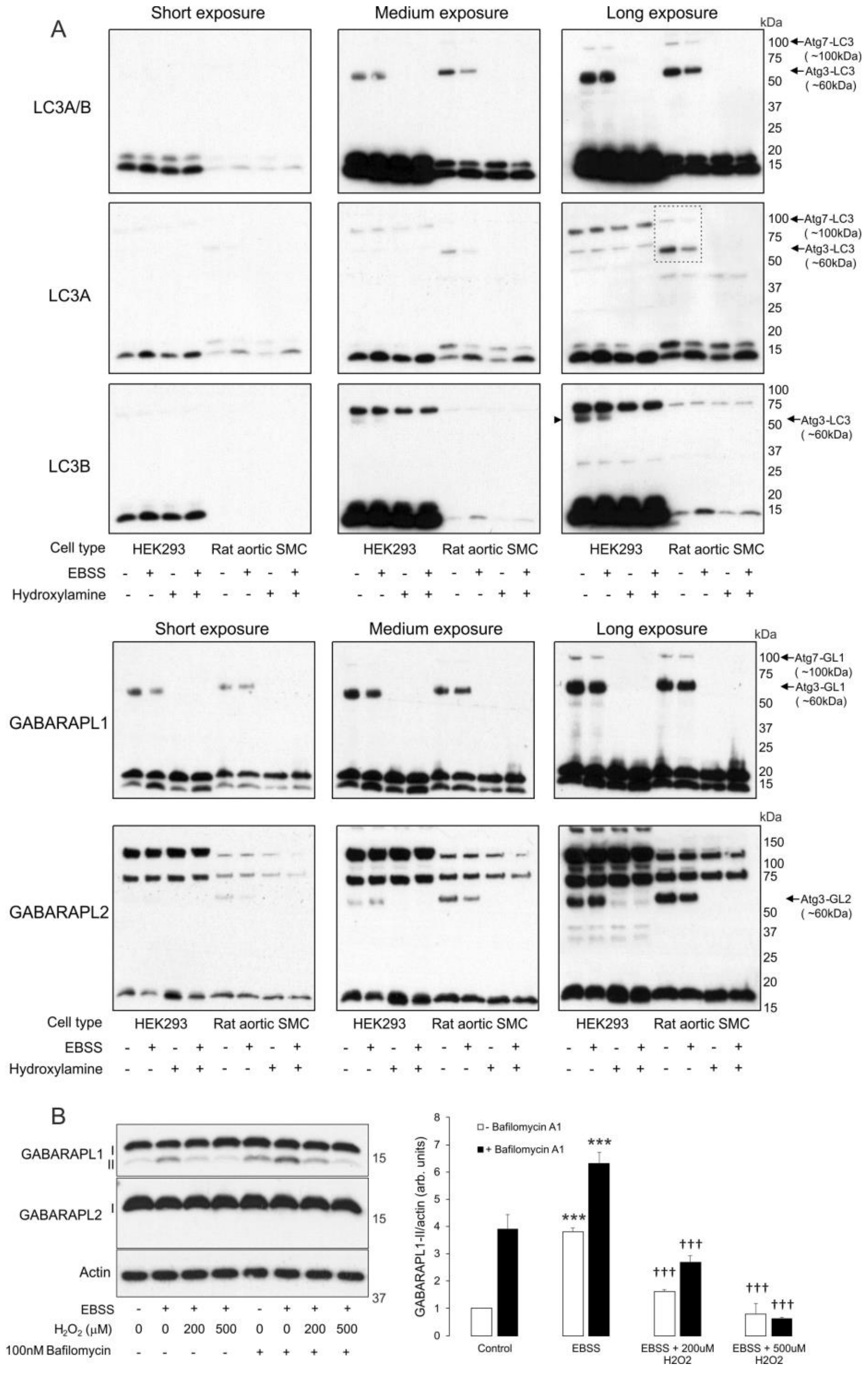

Supplementary Figure 4 In addition to LC3A and B isoforms GABARAPL1 and GABARAPL2 also likely form stable complexes with Atg3 and Atg7. (A) Higher molecular complexes for GABARAPL1 and GABARAPL2 decrease after an hour of amino acid withdrawal in EBSS, and are cleavable with hydroxylamine. The higher molecular complexes for GABARAPL1 and GABARAPL2 are consistent with and at the correct weight for covalent bound Atg3 and Atg7. (B) Amino acid deprivation induced GABARAPL1 lipidation is inhibited by H_2O_2 in the presence or absence of H_2O_2. However, the lipidation of GABRAPL2 could not be detected. The graph to the right shows quantification of GABARAPL1 lipidation in treated HEK cells. All data represent mean ± s.e. from 3 (B) independent experiments. ****P* < 0.005 (Dunnett's test) statistical significance relative to control or bafilomycin A1 alone. †††*P* < 0.005 statistical significance relative to EBSS or EBSS + bafilomycin A1 treatment.

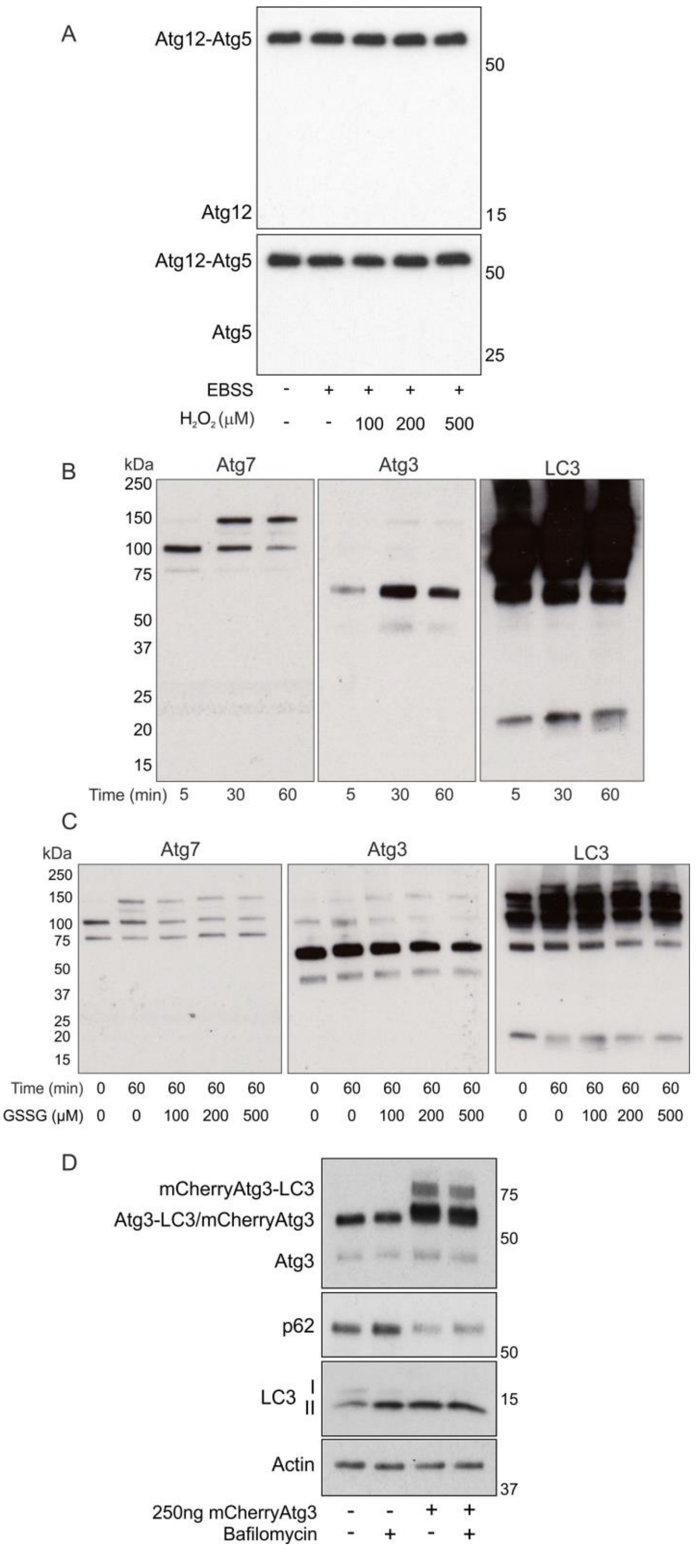

Supplementary Figure 5 Immunoblot analysis of autophagy proteins

(A) Extended immunoblots for Atg5 and Atg12 show they are fully conjugated, which is not impacted by EBSS or H_2O_2 co-treatment. (B) Immunoblot analysis of samples from the activity assay shown in Figure 3B for Atg7, Atg3 and LC3 expression. (C) Immunoblot analysis of samples from the activity assay shown in Figure 3C for Atg7, Atg3 and LC3 expression. (D) Expression of mCherryAtg3 leads to loss in the abundance of p62 and increased LC3 lipidation, consistent with enhanced E2-like enzyme activity.

Figure 1

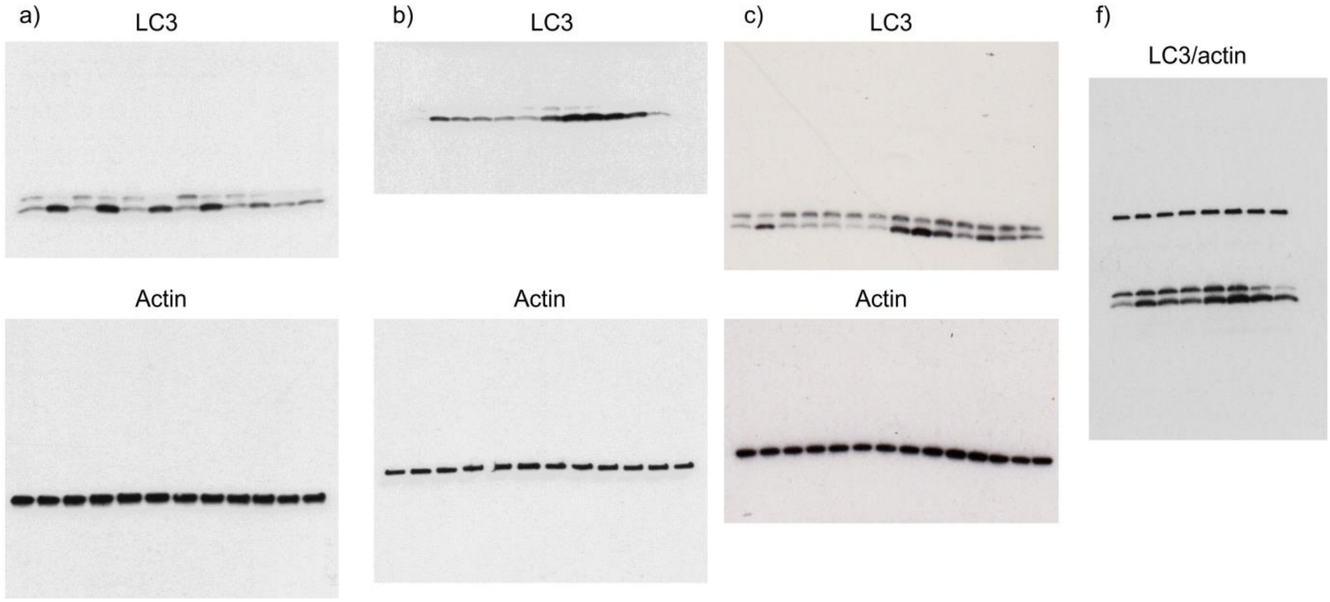

Supplementary Figure 6 Uncropped blots for key figures in the manuscript.

Figure 2

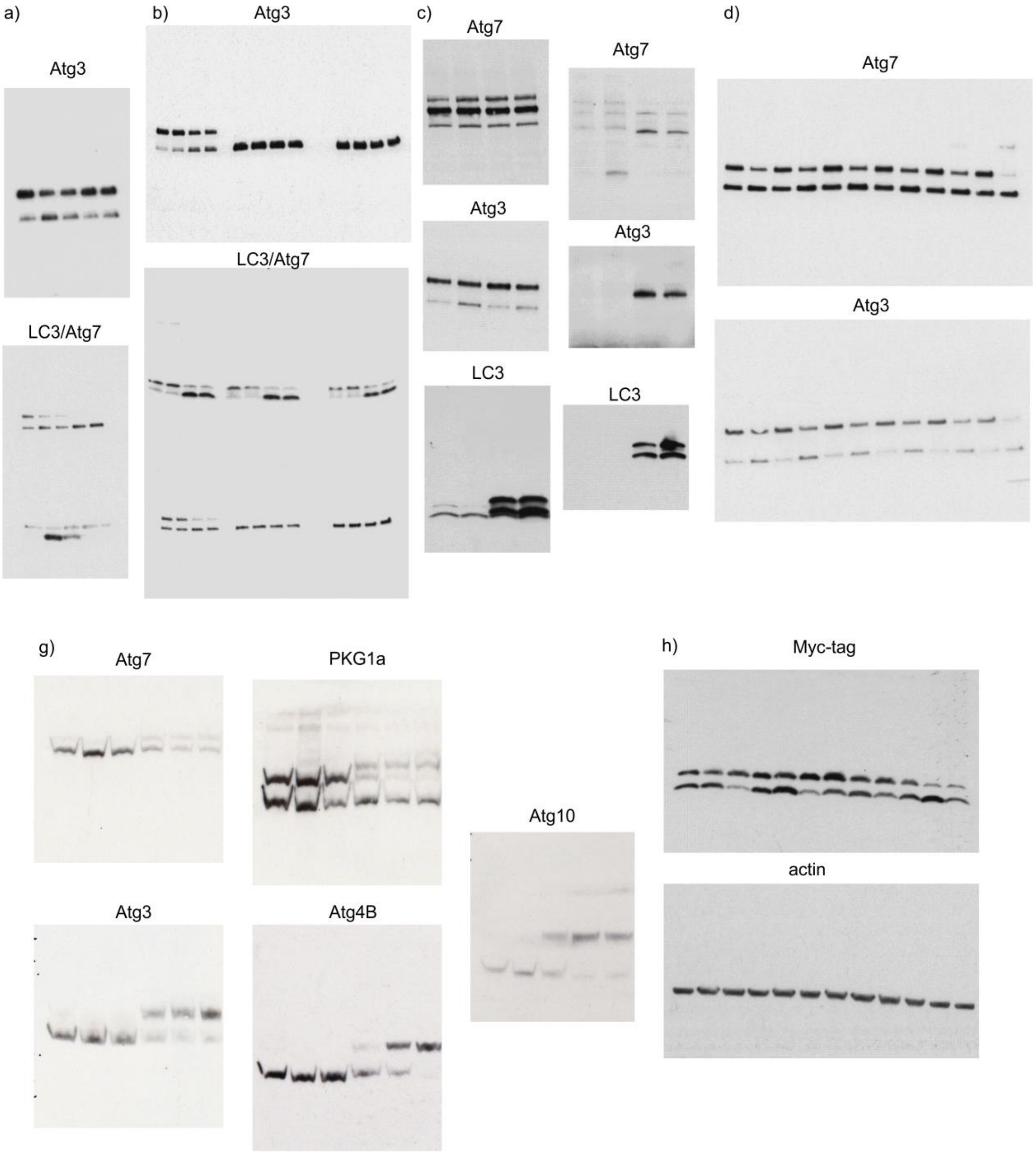
a)
Atg3
LC3/Atg7
b)
Atg3
LC3/Atg7
c)
Atg7
Atg3
LC3
Atg7
Atg3
LC3
d)
Atg7
Atg3
g)
Atg7
PKG1a
Atg3
Atg4B
Atg10
h)
Myc-tag
actin

Figure 4

b) Atg7

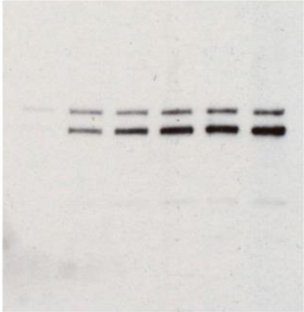

c)

Atg7

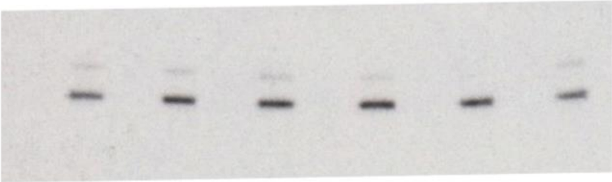

Atg3

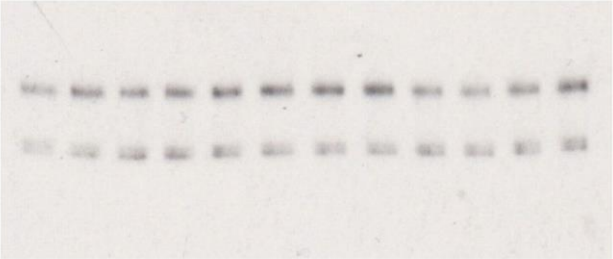

LC3

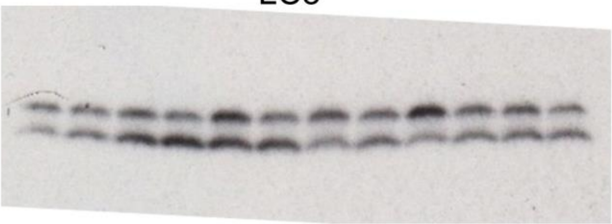

Actin

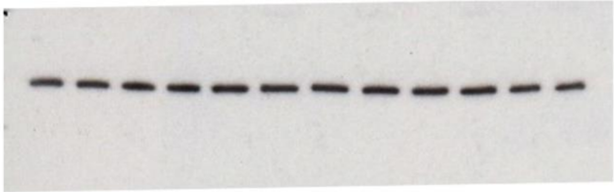

Figure 5

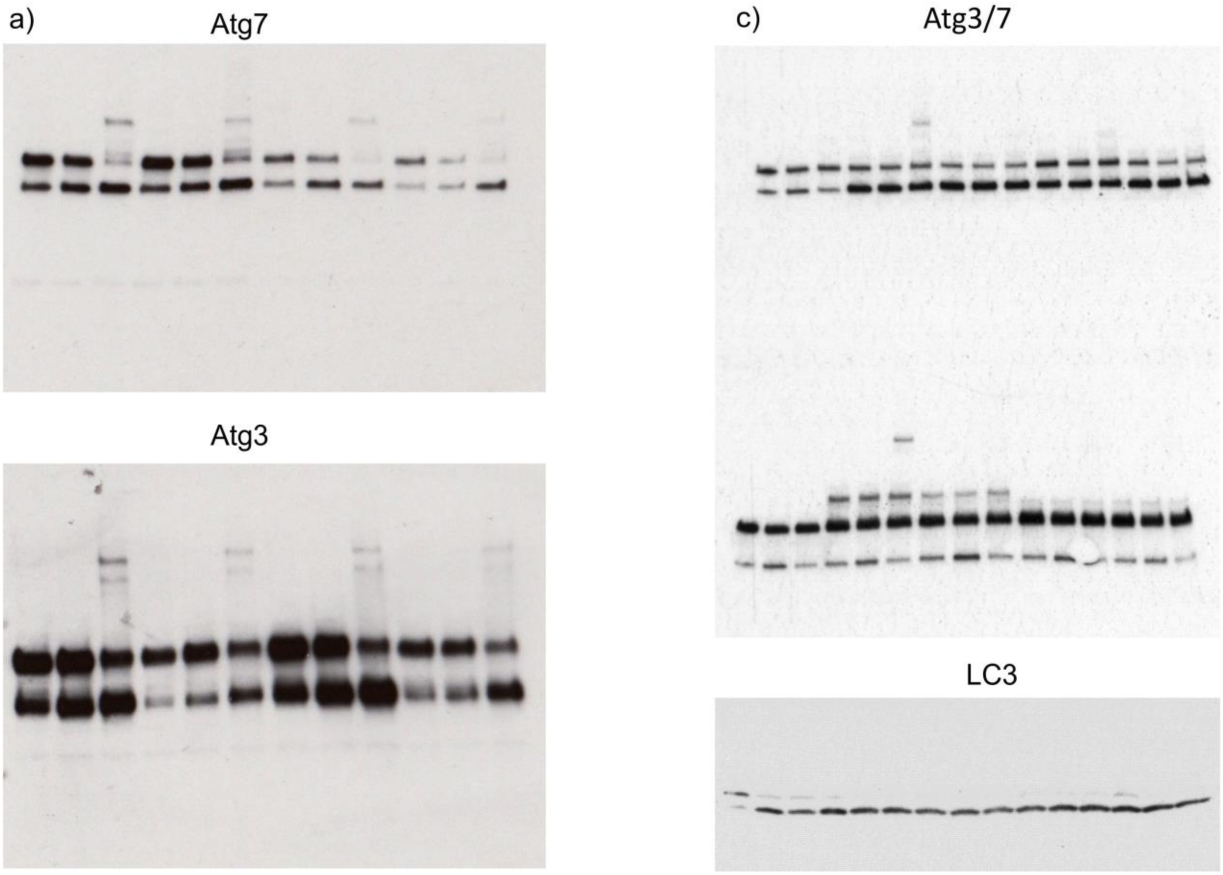

Figure 6

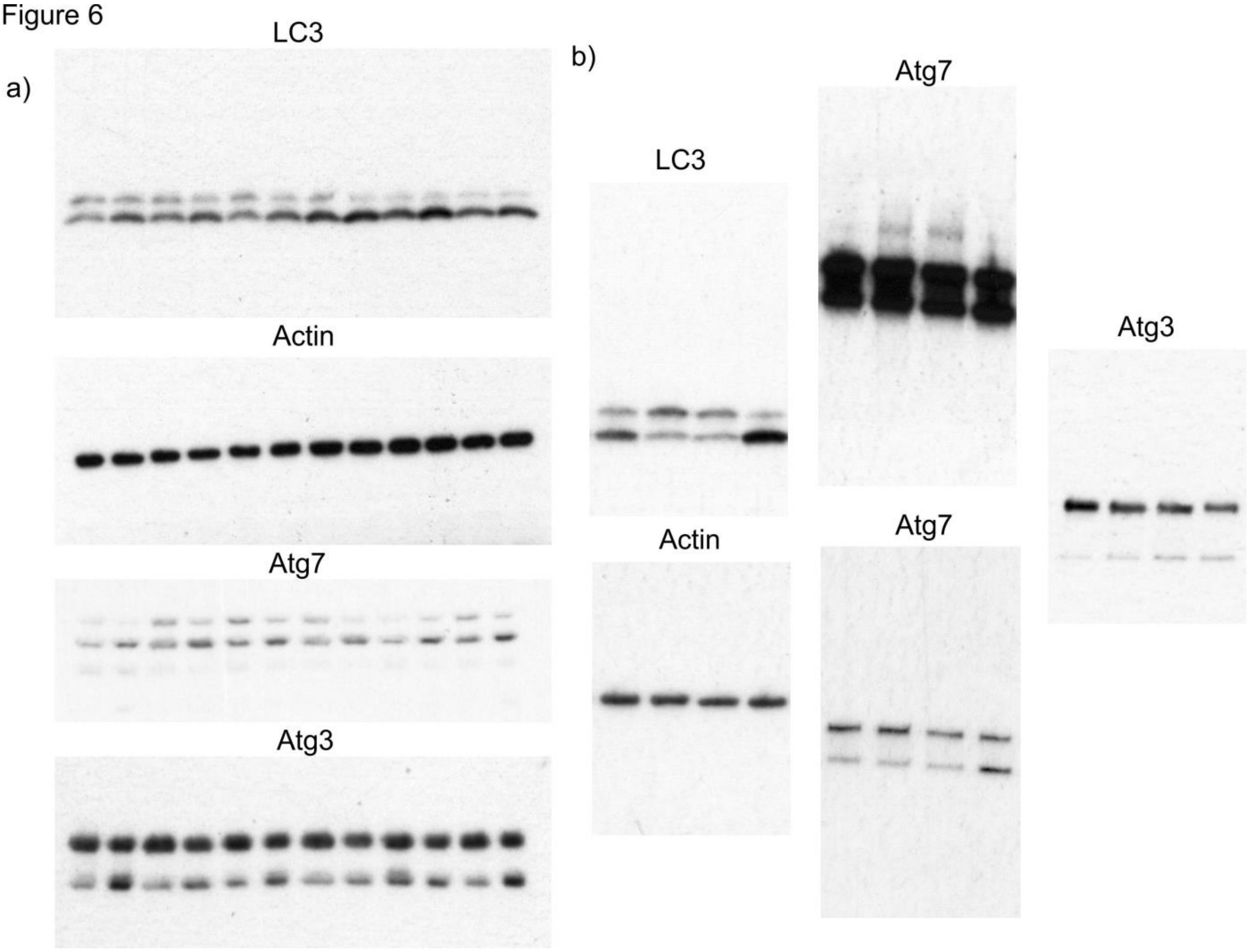

Figure 7

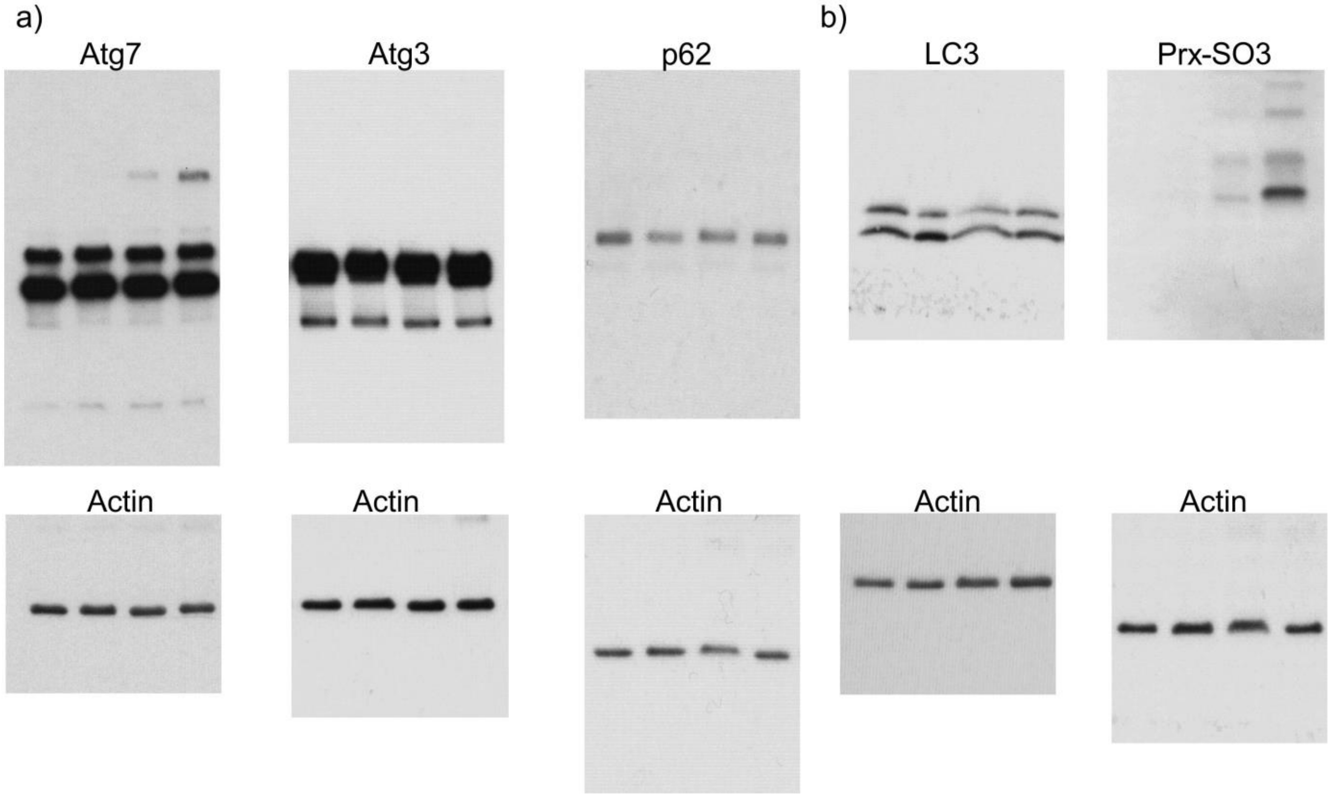

Supplement: Supplementary file 1 — Supplementary Information [file 41467_2017_2352_MOESM1_ESM.pdf]
